# 5mC and H3K9me3 of TRAF3IP2 promoter region accelerates the progression of translocation renal cell carcinoma

**DOI:** 10.1186/s40364-022-00402-3

**Published:** 2022-07-27

**Authors:** Lei Yang, Yi Chen, Ning Liu, Yanwen Lu, Xin Li, Wenliang Ma, Weidong Gan, Dongmei Li

**Affiliations:** 1 Immunology and Reproduction Biology Laboratory & State Key Laboratory of Analytical Chemistry for Life Science, Medical School, Nanjing University, Nanjing, 210093 Jiangsu China; 2 Jiangsu Key Laboratory of Molecular Medicine, Nanjing University, Nanjing, 210093 Jiangsu China; 3 Department of Urology, Affiliated Drum Tower Hospital of Medical, School of Nanjing University, Nanjing, 210008 Jiangsu China; 4 Department of Urology, Nanjing First Hospital, Nanjing Medical University, Nanjing, 210006 Jiangsu China

**Keywords:** TRAF3IP2-AS1, TRAF3IP2, NOTCH1, 5mC, H3K9me3

## Abstract

**Background:**

In our previous study, we found that lncRNA TRAF3IP2 antisense RNA 1 (TRAF3IP2-AS1) could play a critical role in the progression of *NONO-TFE3* translocation renal cell carcinoma (*NONO-TFE3* tRCC). However, the function of TRAF3IP2 (TRAF3 interacting protein 2), encoded by the complementary strand of TRAF3IP2-AS1, remains poorly understood in *NONO-TFE3* tRCC.

**Methods:**

Immunohistochemistry, western blot, and qRT-PCR were undertaken to study the expression and clinical significance of TRAF3IP2 in Xp11.2 tRCC tissues and cells. The functions of TRAF3IP2 in tRCC were investigated by proliferation analysis, EdU staining, colony and sphere formation assay, Transwell assay, and apoptosis analysis. The regulatory mechanisms among TRAF3IP2, NOTCH1, and TRAF3IP2-AS1 were investigated by luciferase assay, RNA immunoprecipitation, western blot, methylated DNA Immunoprecipitation, and CRISPR/dCas9-based system.

**Results:**

The results showed that TRAF3IP2 was highly expressed in *NONO-TFE3* tRCC tissues and cells, and the silence of TRAF3IP2 inhibited the proliferation, migration, and invasion of UOK109 cells which were derived from cancer tissue of patient with *NONO-TFE3* tRCC. Mechanistic studies revealed that TRAF3IP2 functioned as a co-activator of NOTCH1 to activate the NOTCH1 pathway. Meanwhile, HNRNPK, DNMT1 and SETDB1 could be recruited by TRAF3IP2-AS1 to the promoter region of *TRAF3IP2*, which mediated 5-hydroxymethylcytosine (5mC) on DNA and trimethylated lysine 9 of histone H3 (H3K9me3) at transcriptional level to repress the expression of TRAF3IP2.

**Conclusions:**

TRAF3IP2 functions as an oncogene in *NONO-TFE3* tRCC progression and might serve as a novel target for *NONO-TFE3* tRCC therapy.

**Supplementary Information:**

The online version contains supplementary material available at 10.1186/s40364-022-00402-3.

## Introduction

*NONO-TFE3* translocation renal cell carcinoma (*NONO-TFE3* tRCC) is one of the subtypes of Xp11.2 translocation/TFE3 fusion-associated renal cell carcinoma (Xp11.2 tRCC), which is also known as *TFE3*-fusion associated RCC as a new subset of RCC by WHO in 2016 [1, 2]. The typical feature of Xp11.2 tRCC is the high expression of TFE3 fusions caused by the promoter region of partner genes, including *ASPL*, *PRCC*, *NONO*, *CLTC,* and other housekeeping genes [3–5]. More recently, some sequencing efforts revealed that the high expression of TFE3 fusions is the key factor in tumor initiation in Xp11.2 tRCC, which does not harbor any mutations of tumor driver genes [6, 7]. As a transcription factor, wild-type TFE3 regulates gene expression during stress responses [8, 9]. Our previous study reveals that NONO-TFE3 fusion protein contains the DNA binding domain of TFE3 and maintains transcriptional regulation function [10, 11].

In our previous studies, it has been found that TRAF3IP2 (TRAF3 interacting protein 2) is negatively associated with the expression of TRAF3IP2 antisense RNA 1 (TRAF3IP2-AS1) which is down-regulated by NONO-TFE3 fusion [10, 12]. TRAF3IP2 is a crucial regulator of the immune and inflammatory response [13]. As an activator protein in the NF-κB and TNF (tumor necrosis factor), TRAF3IP2 is believed to play a clear role in tumor initiation and progression. In addition to interact with IL-17 protein [14], TRAF3IP2 binds to the mRNA of IL-17 directly to promote inflammation in tumor cells [15]. Recent studies have revealed that TRAF3IP2 promotes glioblastoma growth by enhancing inflammation of the microenvironment and that the silence of TRAF3IP2 inhibits the metastasis and development of breast cancer [16]. The specific biological function of TRAF3IP2 in some cancers is well-defined, but the actual mechanism of TRAF3IP2 in the intracellular component is still unknown.

TRAF3IP2-AS1 is a natural antisense long non-coding RNA (lncRNA) transcribed in the opposite orientation. According to our previous research, low expression of TRAF3IP2-AS1 mediated by NONO-TFE3 fusion promotes the progression of *NONO-TFE3* tRCC. In addition to acting as miRNA sponges, TRAF3IP2-AS1 could interact with the mRNA of PARP1 and promote its decoy by *N*^6^-methyladenosie (m^6^A) modification [12]. Interestingly, the present study indicated the overexpression of TRAF3IP2-AS1 can decrease the expression of TRAF3IP2. However, the correlation between TRAF3IP2 and TRAF3IP-AS1 is poorly defined.

As a kind of multifunctional molecule, lncRNAs play essential roles in tumorigenesis and progression [17, 18]. It is widely known that lncRNAs act as a molecular 'sponge' to absorb small RNAs, such as microRNAs [19] and PIWI-interacting RNAs (piRNAs) [20], to form competing endogenous RNA (ceRNA) network and mediate the expression of target genes, even some oncogenes or tumor suppressor genes. The direct interaction between lncRNAs and mRNAs [21, 22], which could regulate mRNA stability and translation efficiency, brings new surprises to lncRNA-based therapeutics.

As a class of RNA binding proteins, HNRNPs could mediate RNA processing by binding to RNA, including RNA splicing, transcription, translation, and degradation. Increasing evidence suggests that HNRNPs have been strongly implicated in tumor initiation and progression. HNRNPA1 promotes the advancement of oral squamous cell carcinoma by selective shearing the pre-mRNA of cyclin-dependent kinase 2 [23]. In different human tumors, HNRNPC exhibits other biological functions. HNRNPC binds to pri-miR-21 and activates the AKT pathway to promote the capacity of migration and invasion of glioblastomas [24], but HNRNPC enhances the apoptosis of ovarian cancer through binding to miR-744 [25]. Interestingly, HNRNA2B1 and HNRNPC could function as m6A readers during the processing of RNA modification [26].

In the present study, we found that overexpressed TRAF3IP2 in *NONO-TFE3* tRCC could enhance tumor progression through function as a co-activator of NOTCH1 to mediate NOTCH1 target genes. We discovered that TRAF3IP2-AS1 could bind to the promoter region of *TRAF3IP2* and recruit HNRNPK, DNMT1, and SETDB1 to repress the expression of TRAF3IP2 via mediating 5-hydroxymethylcytosine (5mC) on DNA and trimethylated lysine 9 of histone H3 (H3K9me3). In essence, our data indicate that TRAF3IP2 is a tumor-derived factor that is important for tumor progression in *NONO-TFE3* tRCC, uncovering a new regulatory mechanism that drives tumor progression.

## Materials and methods

### Cell culture and tissue samples

A number of cell lines were obtained from Type Culture Collection of Chinese Academy of Sciences (Shanghai, China). In contrast, the Xp11.2 tRCC cell lines UOK120 and UOK109 were kind gifts from Dr. W. Marston Linehan (National Cancer Institute, Bethesda, MD). According to the description, two cell lines derived from primary papillary cell carcinomas were generated, including UOK120 (PRCC-TFE3 fusion) and UOK109 (NONO-TFE3 fusion) originating in a 30-year-old male and a 39-year-old male, respectively. DMEM (WISENT, St Bruno, Quebec, Canada) supplemented with 10% FBS (Excell, Nanjing, China) and 1% penicillin–streptomycin (WISENT) was applied for cell culture. At 37 °C, the cells were cultured under a standard humidified environment containing 5% CO_2_.

An experienced pathologist confirmed each sample collected from Nanjing Drum Tower Hospital (Department of Pathology, Nanjing Drum Tower Hospital). During their consultations, all patients were informed that their tissue would be used for scientific research and signed the consent form. The study was approved by the Medical Ethics Committee of Affiliated Drum Tower Hospital of Medical School of Nanjing University.

### RNA isolation and quantitative real-time PCR (qRT-PCR) assays

Isolation of total RNA was performed using RNA isolater Total RNA Extraction Reagent (R401, Vazyme biotech Co., Ltd, Nanjing, China), as per the product description. To reverse transcript RNA into cDNA, Hiscript Q RT qPCR Supermix (R122, Vazyme) was used, then qRT-PCR assay was applied to quantify the cDNA, and ChamQ SYBR qPCR Master Mix (Q712, Vazyme) was used to acquire the data. Normalization was performed using 18 s rRNA as an internal control. As shown in Table S1, primers for RNAs were designed.

### Chromatin immunoprecipitation (ChIP) assay, dCas9-ChIP assay, and methylated DNA Immunoprecipitation (MeDIP) assay

The ChIP assay and the dCas9-ChIP assay were performed according to the Pierce™ Agarose ChIP Kit (Thermo Scientific, Carlsbad, CA), and the MeDIP assay was performed according to the manufacturer's protocol of the MeDIP kit (BersinBio, Guangzhou, China). After fixing, lysing, and sonicating the cells to prepare fragments, specific antibodies were used overnight to precipitate the chromatin. The binding complex is then thoroughly washed, extracted, purified, and analyzed by qRT-PCR or western blot. Table S2 contains primers.

### Dual-luciferase reporter assay

Transfection of the plasmids or lentivirus was performed in HEK293T cells. Dual-Luciferase Reporter Assay Kit (DL101, Vazyme) was used to measure firefly luciferase and Renilla luciferase activity in each cell, and Renilla was used as an internal control.

### RNA immunoprecipitation (RIP) and Chromatin Isolation by RNA Purification (ChIRP)

The RIP assays were carried out according to the Millipore Magna RIP Kit (Millipore, Darmstadt, Germany). Anti-IgG/HNRNPK-conjugated beads were incubated with the indicated cells lysis in RIP lysis buffer overnight at 4 °C. The binding complex is then thoroughly washed, extracted, purified, and analyzed by qRT-PCR or western blot. For MS2-RIP, a different kind of truncation plasmid combined with GFP-MS2 was transfected into HEK293T cells, and the cell lysis was incubated with anti-GFP -conjugated beads. The procedure of ChIRP was carried out according to the manufacturer's instructions for a ChIRP kit (BersinBio). Briefly, the prepared lysis was enriched using the probe. Then, the prepared lysis was enriched with the probe, and then the antigen-binding complexes were thoroughly washed, eluted, purified, and then analyzed with real-time PCR or western blot.

### Nuclear and cytoplasmic protein extraction

According to the instructions provided by the manufacturer, nuclear material was extracted using a Nuclear and Cytoplasmic Protein Extraction Kit (Beyotime, Shanghai, China). Centrifugation was performed after cells were suspended in a cytoplasmic extraction reagent. A supernatant of the cytoplasmic extract was obtained by centrifuging the pellet after it was suspended with nuclear extraction reagent and subjected to centrifuging for the supernatant, which represents nuclear extract. By western blot, the nuclear extract and the cytoplasmic extract were analyzed.

### Western blot

Following indicated treatments, such as transfection with lentivirus or administration of small molecule, total protein was isolated from cells. The lysate was prepared from an ice-cold extraction buffer. In the following step, soluble fractions were mixed with 5 × loading buffer and heated at 100 °C for five minutes. Standard procedures were applied to separate proteins using SDS-PAGE and PVDF membranes (Roche, Basel, Switzerland). For one hour, the blocks were blocked in 5% nonfat milk at room temperature. The 3% BSA solution (Sigma Aldrich) was used as an overnight incubation medium for primary antibodies. The secondary antibodies were incubated for 1 h at room temperature with HRP-conjugated primary antibodies. The proteins were identified with Millipore's ECL solution, and their intensity was quantified with Image J software from the National Institutes of Health. As an internal control, ACTB has also been chosen. A description of the antibody can be found in Table S3.

### Flow cytometry

According to the manufacturers' protocol, flow cytometry was carried out. The cells were then incubated with reagents from an Annexin V-FITC / propidium iodide (PI) apoptosis kit (Vazyme) and analyzed using a BD Beckman cytometer (BD Biosciences) and FlowJo software. In order to analyze the cell cycle, PI / RNase staining kit (BD Biosciences) reagents were used.

### CCK8, 5-Ethyny-2’-deoxyuridine (EdU) assay, clone forming, and sphere formation

Cell proliferation assays were performed using the Vazyme Cell Counting Kit 8 (CCK8; Vazyme). EdU (Beyotime, Shanghai, China) according to the manufacturer's protocol. The clone-forming capacity of transfected cells was assessed on 6-well plates with 500 cells / well over a period of two weeks. The sphere formation assay was performed using 96-well plates with Ultra Low Attachment (Corning, NY, cat. no. 174925). Two weeks later, pictures of the spheres were taken.

### Transwell assay

The Transwell technique was used to evaluate cell migration and invasion. Polycarbonate inserts (Millipore) were used for migration, while BioCoatTM inserts (BD Biosciences) were used for invasion. 200 μL FBS-free medium containing 1 ~ 5 × 10^4^ cells was added to the upper chamber, and 500 μL of DMEM containing 10% FBS was added to the lower chamber. 200 μL FBS-free medium containing 1 ~ 5 × 10^4^ cells was added into the upper chamber, with 500 μL DMEM containing 10% FBS added into the lower chamber. Under a microscope, the stained cells were counted and analyzed after crystal violet staining.

### Immunohistochemistry

Two samples of *NONO-TFE3* tRCC and three samples of ccRCC were taken for immunohistochemistry. Firstly, paraffin-embedded sections were deparaffinized and incubated at 4 °C overnight with rabbit polyclonal anti-TRAF3IP2 (Abclonal, Wuhan, China) primary antibodies. Sections were washed three times and incubated with HRP conjugated goat anti-mouse secondary antibody. After washing the sections three times, the DAB Substrate kit was used to detect the signal following the manufacturer's instructions.

### Co-immunoprecipitation (CoIP) assay

CoIP was performed following the manufacturer's instructions (BersinBio). Lysis buffer supplemented with protease inhibitors was used to collect and lyse the cells, and the lysate was centrifuged to clear. An overnight immunoprecipitation process was done with protein lysate and primary antibody. The precipitants were extensively washed with wash buffer, boiled with 5 × loading buffer, and subjected to western blot.

### Methylation-specific PCR (MSP)

The genomic DNA was extracted with phenol–chloroform. During 16 h at 55 °C, genomic DNA was modified with bisulfite (Sigma, Shanghai, China). qRT-PCR was used to analyze samples after purification, neutralization, ethanol precipitation, drying, resuspension, and resuspension. PCR primers are listed in Table S4.

### Small interfering RNA (siRNA), short hairpin RNA (shRNA), antisense oligonucleotides (ASOs), small molecule inhibitors, and cell transfection

Synthesized siRNA was produced by Gene Pharma (Suzhou, China), and ASOs were produced by RiboBio (Guangzhou, China). The sequence of shRNA was obtained from MISSION shRNA (Sigma-Aldrich) and synthesized by Tsingke (Nanjing, China). Then, the fragment was inserted into the pLVX-shRNA1 vector by 5 min Universal Ligation Mix (Vazyme). The information on small molecule inhibitors is provided in Table S5. The lentiviruses were made by OBiO Technology (Shanghai, China). Transfection was performed using LipoFiter 3.0 (Hanbio, Shanghai, China) according to the manufacturer's instructions. 48 h after transfection, cells were harvested. The sequences are provided in Table S6.

### CRISPR/Cas9-based system design

To deliver the exogenously expressed protein to the promoter of TRAF3IP2, we designed a system based on CRISPR/Cas9. The candidate proteins (HNRNPK/DNMT1/SETDB1) were fused to the MS2 binding protein, and the nuclear localization signal (NSL) was added to the C-terminus of inactive Cas9 (dCas9). The gRNA expression box containing the MS2 stem-loop was subcloned from LentiSAM V2 (Puro) by ClonExpress Ultra One Step Cloning Kit (C115, Vazyme). Thus, the CRISPR-Cas9 complex could recruit the MS2-HNRNPK, MS2-DNMT1, or MS2-SETDB1 by the MS2 stem-loop to the promoter of TRAF3IP2. GeneChem Technology constructed the Synergistic Activation Mediator (SAM) system based on CRISPR/Cas9. The sequences of gRNA are provided in Table S7.

### Plasmid construction, lentivirus package, and infection

The candidate proteins TRAF3IP2/NOTCH1/HNRNPK/SETDB1/DNMT1 were amplified and fused to tag protein (HA/Flag/V5) and subcloned into pCDH-EF1-MCS-PGK-Puro. pcDNA3.1 and pSL-MS2-12x (Addgene, Cambridge, MA) were used for cloning the TRAF3IP2-AS1 sequence generated by GeneChem Technology (Shanghai, China). The fragment of these proteins was amplified from these full-length plasmids and subcloned into pCDH-EF1-MCS-PGK-Puro or pSL-MS2-12x. Lentivirus particles were produced by transfection of HEK293T cells with 4 μg of psPAX2, 2 μg of pMD2.G, and 5 μg of shRNA construct or overexpression vector.

The resulting supernatant containing lentivirus particles were collected after 48 and 72 h, and target cells were infected with these viruses in the presence of 8 mg/mL polybrene. During the first 24 h following transfection, treatments were administered. After 72 h of transduction, cells were selected with puromycin for four days. The sequences of primers are provided in Table S8.

### Animal experiment

BALB/c mice six weeks old were selected for xenograft experiments and maintained in special pathogen-free conditions. Under animal protocol number SYXK (Su) 2009–0017, all procedures were approved by the Animal Care and Use Committee of Nanjing University. Mice were injected subcutaneously with shNC/shTRAF3IP2 lentiviral constructs (5 × 10^6^, 200uL) transfected with 786-O cells. Experiments were performed in groups of four mice. Mice were sacrificed after 50 days, and the tumor volume was calculated using the following formula (volume (cm^3^) = [width^2^ (cm^2^) × length (cm)]/^2^).

### Statistical analysis

All calculations were performed by GraphPad Prism 8.0 (GraphPad Software, San Diego, CA). Student's t-tests and one-way analysis of variance (ANOVA) were used to assess the significance of differences. *P* < 0.05 was considered statistically significance (*P < 0.05, ** *P* < 0.01, and *** *P* < 0.001). All values are expressed as the means ± standard deviation.

## Results

### TRAF3IP2 is overexpressed in *NONO-TFE3* tRCC and critical for malignant phenotypes

**Fig. 1:**
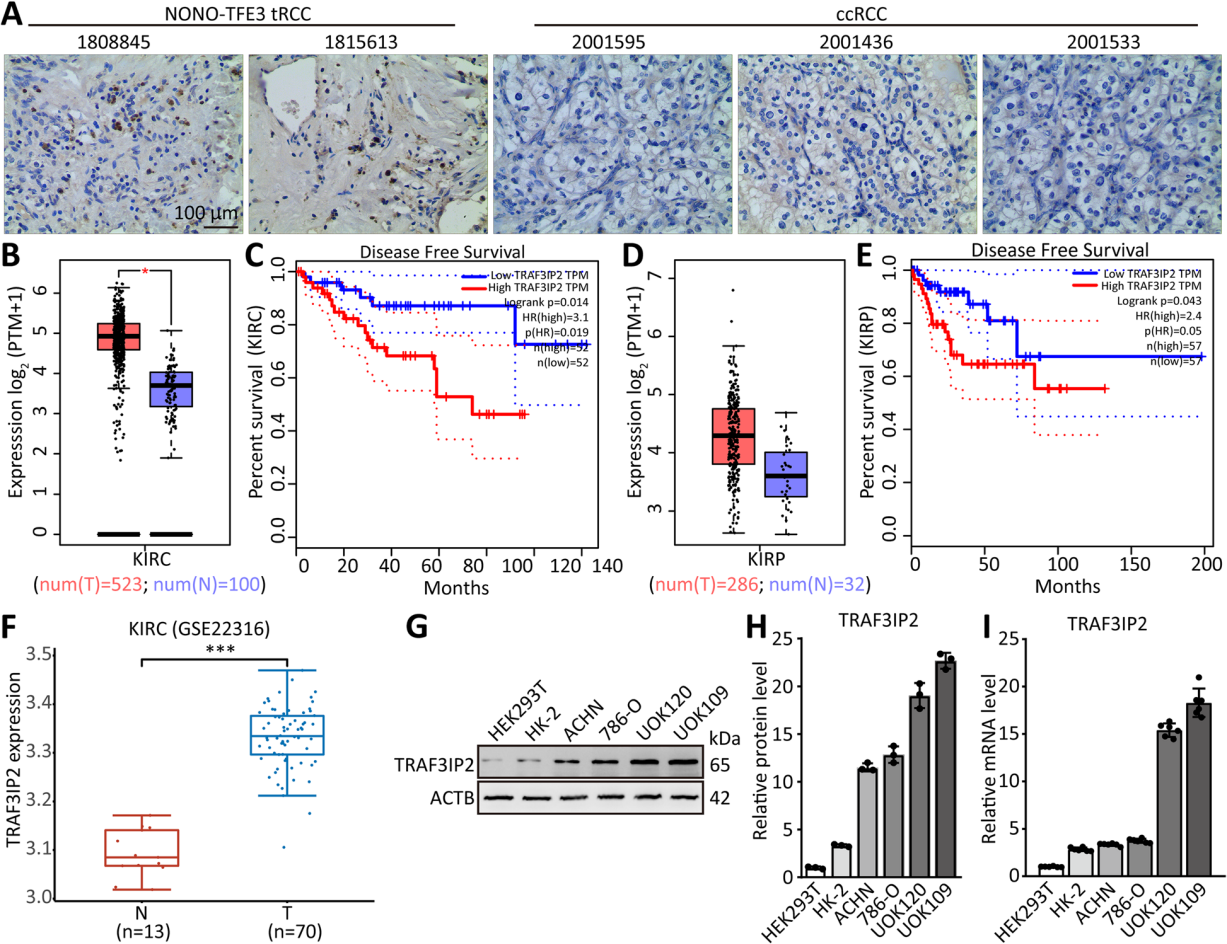
TRAF3IP2 is overexpressed in *NONO-TFE3* tRCC and critical for malignant phenotypes. **A** The protein level of TRAF3IP2 was analyzed by immunohistochemistry in *NONO-TFE3* tRCC and ccRCC. **B, D** Analysis of TRAF3IP2 in KIRC and KIRP tissues compared with normal tissues were performed using TCGA data. **C, E** Kaplan–Meier analysis revealed the disease-free survival (DFS) in KIRC patients based on the relative TRAF3IP2 expression. **F** Analysis of TRAF3IP2 in ccRCC tissues compared with normal tissues was performed using GEO data (GSE22316). **G-I** The protein and mRNA levels of TRAF3IP2 was analyzed by qRT-PCR and western blot assay in ccRCC cell line (786-O and ACHN), tRCC cell lines (UOK109 and UOK120) and normal renal cell lines (HK-2 and HEK 293 T). The data are presented as the mean ± SD, *P < 0.05, ***P < 0.001

Since a lack of evidence has been found on the role of TRAF3IP2 in cancer, we first determined its clinical implication by comparing the expression of TRAF3IP2 in both *NONO-TFE3* tRCC and clear cell renal cell carcinoma (ccRCC). The result of immunohistochemistry showed that the expression of TRAF3IP2 was higher in *NONO-TFE3* tRCC than in ccRCC (Fig. 1A). The TCGA database was applied to investigate the relationships between TRAF3IP2 expression and the patient's prognosis. The results showed a significant correlation between high expression of TRAF3IP2 and poor prognosis in kidney renal clear cell carcinoma (KIRC) and kidney renal papillary cell carcinoma (KIRP; Fig. 1B-E). KIRC tumor groups expressed higher levels of TRAF3IP2 than normal groups, according to the GEO database (GSE22316, Fig. 1F).

**Fig. 2:**
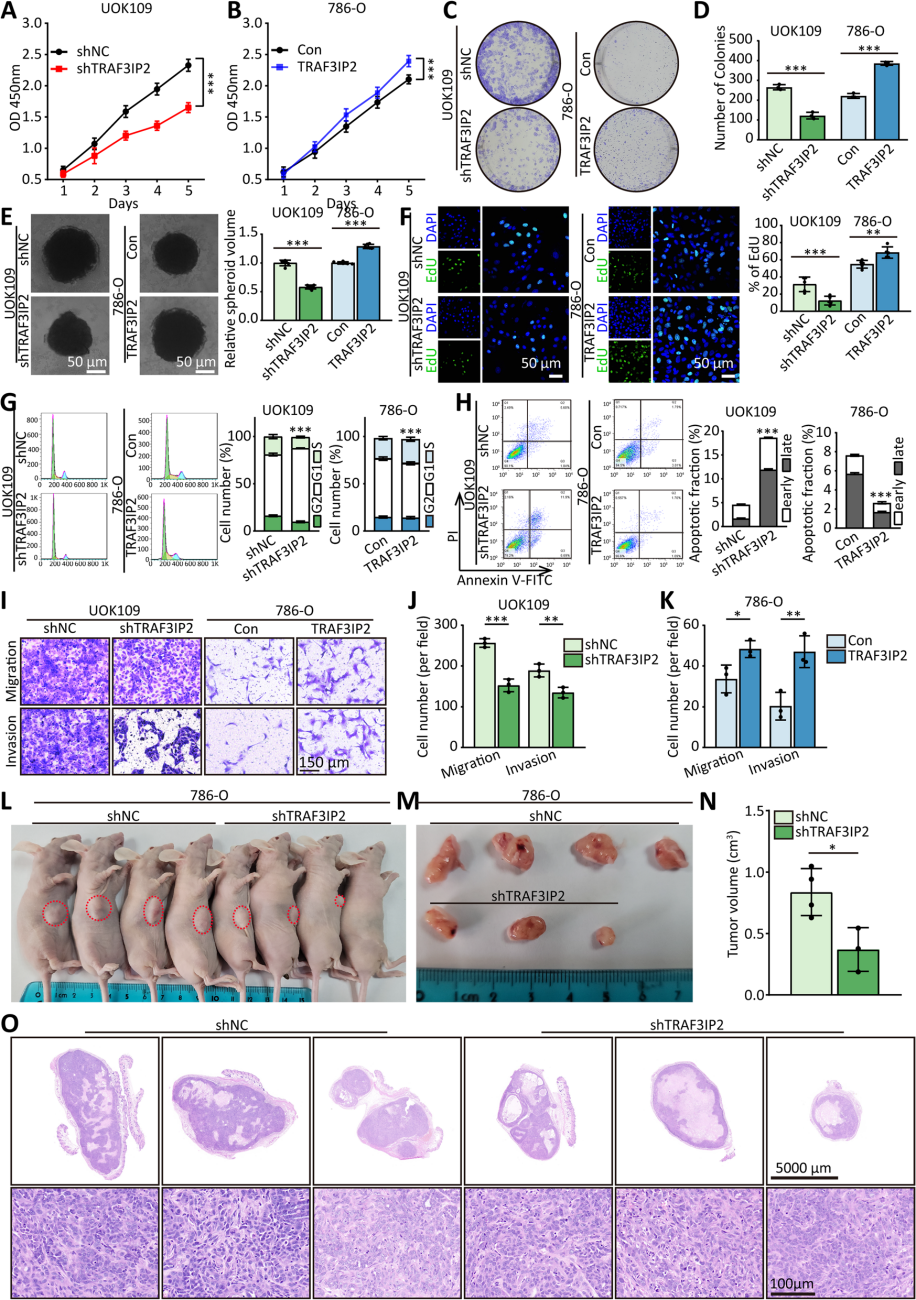
High expression of TRAF3IP2 induces development of *NONO-TFE3* tRCC. **A-E** The effects of TRAF3IP2 overexpression or knockdown on the proliferation of UOK109 and 786-O cells respectively were examined by CCK-8 assay (**A-B**), colony formation assays (**C-D**) and tumor sphere formation (**E**). **F** EdU assays were used to detect the proliferation rate of UOK109 and 786-O cells after transfection for 48 h. **G** Cell cycle was analyzed using flow cytometry after transfection for 48 h. The statistical results of the proportion of cells in S stage in correspond groups. **H** Cell apoptosis was analyzed via flow cytometry using an Annexin V/PI kit after transfection for 48 h. **I-K** Migration and invasion assays were performed with transfected cells using Transwell inserts. **L-M** Nude mice were injected subcutaneously with 786-O cells and tumor formation monitored over a period of several weeks. **N** The tumor volume was measured as indicated. **O** Representative H&E staining of xenograft tumors. The data are presented as the mean ± SD, *P < 0.05, **P < 0.01, ***P < 0.001

To find out whether TRAF3IP2 has involved in *NONO-TFE3* tRCC progression, we examined the expression levels of the protein in several RCC cell lines and normal cells. It was observed that TRAF3IP2 expressed the lowest levels in HEK293T cells (Fig. 1G-I), whereas the highest expression was found in the UOK109 cell line, which was derived from cancerous tissues of a patient with *NONO-TFE3* tRCC. To further explore the biological function of TRAF3IP2 in *NONO-TFE3* tRCC, we downregulated the TRAF3IP2 in UOK109 cells and upregulated it in 786-O cells (Figure S1A), then CCK-8 (Figs. 2A and B, S1B), colony formation (Fig. 2C and D), tumorsphere formation (Fig. 2E), EdU (Fig. 2F), flow cytometry (Fig. 2G) and Transwell (Fig. 2I-K) assays were performed. Compared to the negative control, shTRAF3IP2 inhibited cell proliferation, cell cycle, migration, and invasion in UOK109 cells., and this behavior is enhanced by overexpression of TRAF3IP2 in 786-O cells. TRAF3IP2 has been demonstrated to increase apoptosis of UOK109 cells when silenced, and overexpression of TRAF3IP2 reduces apoptosis of 786-O cells when overexpressed (Fig. 2H). Correspondingly, the silence of TRAF3IP2 inhibited tumor growth in vivo (Fig. 2L-O). Overall, TRAF3IP2 could promote the progression of *NONO-TFE3* tRCC.

### TRAF3IP2 promotes the progression of *NONO-TFE3* tRCC by activating the NOTCH signaling pathway

**Fig. 3:**
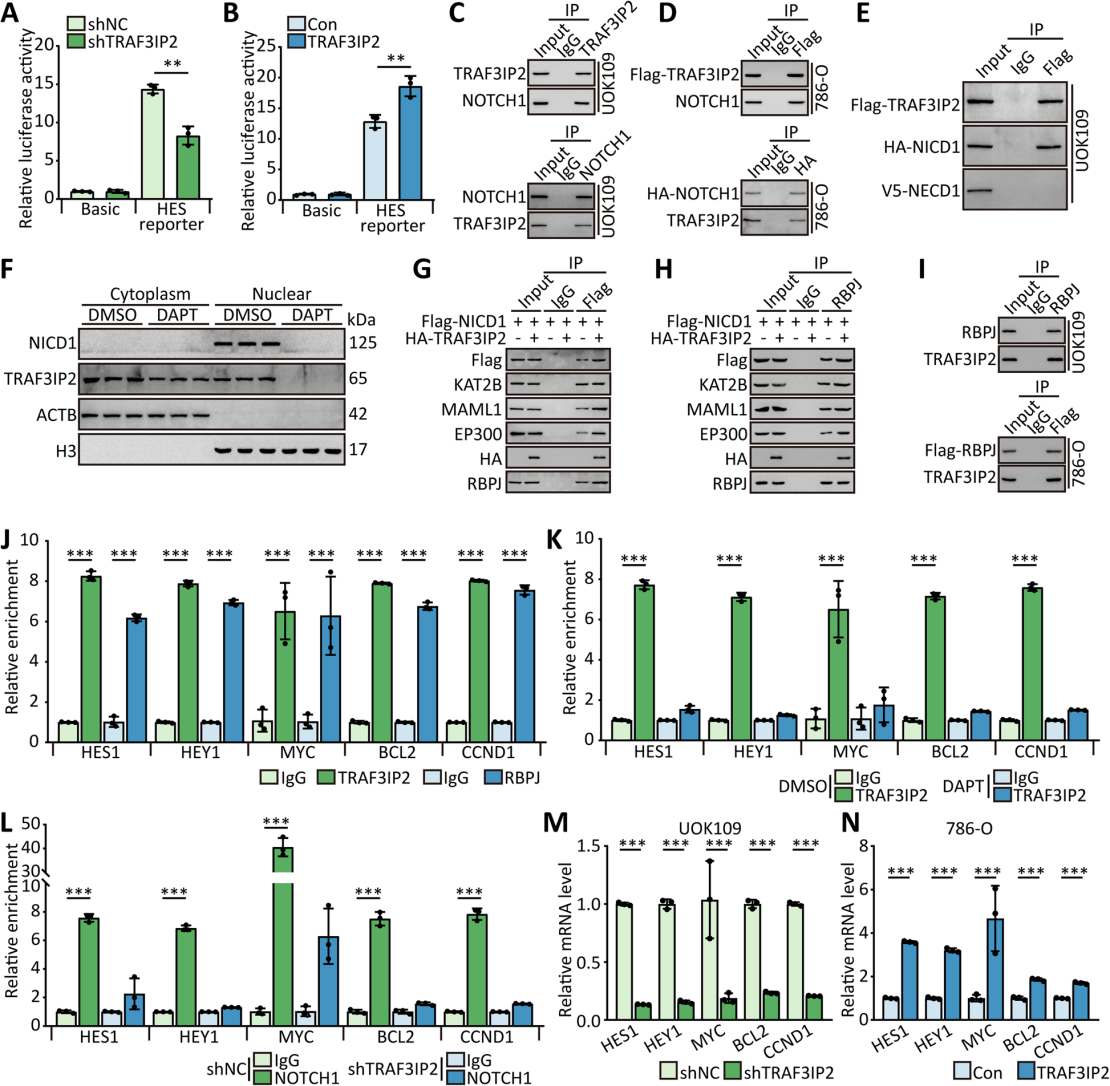
TRAF3IP2 promotes the progression of *NONO-TFE3* tRCC by activating NOTCH signaling pathway. **A-B** HEK 293 T cells were co-transfected with TRAF3IP2 plasmid and HES1 reporter plasmid, and the luciferase activity was determined using a dual luciferase reporter assay after 48 h. **C** UOK109 cells were lysed, then CoIP and reverse CoIP were performed with anti-TRAF3IP2 antibody, anti-NOTCH1 antibody or normal rabbit IgG. **D** 786-O cells were transfected with Flag-TRAF3IP2 or HA-NOTCH1, then CoIP assay was performed with anti-Flag antibody, anti-HA antibody or normal rabbit IgG. **E** UOK109 cells were transfected with Flag-TRAF3IP2, HA-NICD1 and V5-NECD1, then CoIP assay was performed with anti-Flag antibody. **F** Nuclear and cytosolic extracts of UOK109 cells were isolated after treatment with DAPT, and the nuclear translocation of TRAF3IP2 and NICD1 were analyzed by western blot. **G-I** UOK109 or 786-O cells were transfected with indicated lentivirus, then CoIP assay was performed with anti-Flag antibody or anti-RBPJ antibody. **J** ChIP assays showed TRAF3IP2 and RBPJ binding to the promoter region of NOTCH1 target genes. **K-L** UOK109 cells were treated with DAPT or transfected with shTRAF3IP2, then ChIP assay was performed with anti-TRAF3IP2 antibody or anti-NOTCH1 antibody. **M–N** The mRNA level of target genes of NOTCH1 was detected in UOK109 cells and 786-O cells transfected indicated lentivirus. The data are presented as the mean ± SD, *P < 0.05, **P < 0.01, ***P < 0.001

Since it has been reported that TRAF3IP2 affected the activation of the NOTCH1 signaling pathway directly in oligodendrocyte progenitor cells [27], a reporter plasmid of HES1, a downstream target of NOTCH1, was constructed to validate it in UOK109 cells. The result of the luciferase assay showed that downregulation of TRAF3IP2 inhibited the luciferase activity of HES1 reporter in UOK109 cells (Fig. 3A), and higher luciferase activity was observed in TRAF3IP2-overexpressed 786-O cells (Fig. 3B), which confirmed that TRAF3IP2 could activate the NOTCH1 signaling pathway. Furthermore, the result of the CoIP assay showed that TRAF3IP2 could interact with NOTCH1 in UOK109 and 786-O cells (Fig. 3C and D). Furthermore, TRAF3IP2 could interact with NOTCH1 via the intracellular domain (NICD1) but not the extracellular domain (NECD1, Fig. 3E).

The result of nuclear-cytosol extraction isolation showed that the treatment of DAPT, an inhibitor of NOTCH1 cleavage, inhibited TRAF3IP2 nuclear translocation (Fig. 3F). When NOTCH1 functions as a transcription factor, RBPJ, MAML1, EP300, and KAT2B are recruited to NICD1 and form a complex [27]. An interesting finding was that TRAF3IP2 was found in the RBPJ-NICD1 complex, and overexpression of TRAF3IP2 caused MAML1, EP300, and KAT2B to become involved in the TRAF3IP2-NICD1-RBPJ complex (Fig. 3G-I), which indicated that TRAF3IP2 could affect the activation of NOTCH1 pathway. The data of ChIP was consistent with the CoIP result, TRAF3IP2 was enriched at the promoter region of NOTCH1 target genes (Fig. 3J). Meanwhile, the treatment of DAPT abolished the enrichment of NICD1 and TRAF3IP2, like shTRAF3IP2 (Fig. 3K-L). Correspondingly, the mRNA level of HEY1, HES1, MYC, BCL2, and CCND1 were tested to validate the above results (Fig. 3M and N).

CCK-8, EdU, flow cytometry, and Transwell assays were performed to investigate the biological function of NOTCH1 in the *NONO-TFE3* tRCC. Cell proliferation, cell cycle, migration, and invasion were inhibited by shNOTCH1 compared with the negative control in UOK109 cells, and overexpression of NICD1 enhanced these behaviors of 786-O cells (Figure S2A-G and S2I-K). Flow cytometric experiments showed that down-regulation of NOTCH1 obviously increased the apoptosis in UOK109 cells and vice versa (Figure S2H). Collectively, TRAF3IP2 acts as an activator of the NOTCH1 signaling pathway to mediate the progression of *NONO-TFE3* tRCC.

### TRAF3IP2-AS1 down-regulates TRAF3IP2 by recruiting HNRNPK to *TRAF3IP2* promoter

**Fig. 4:**
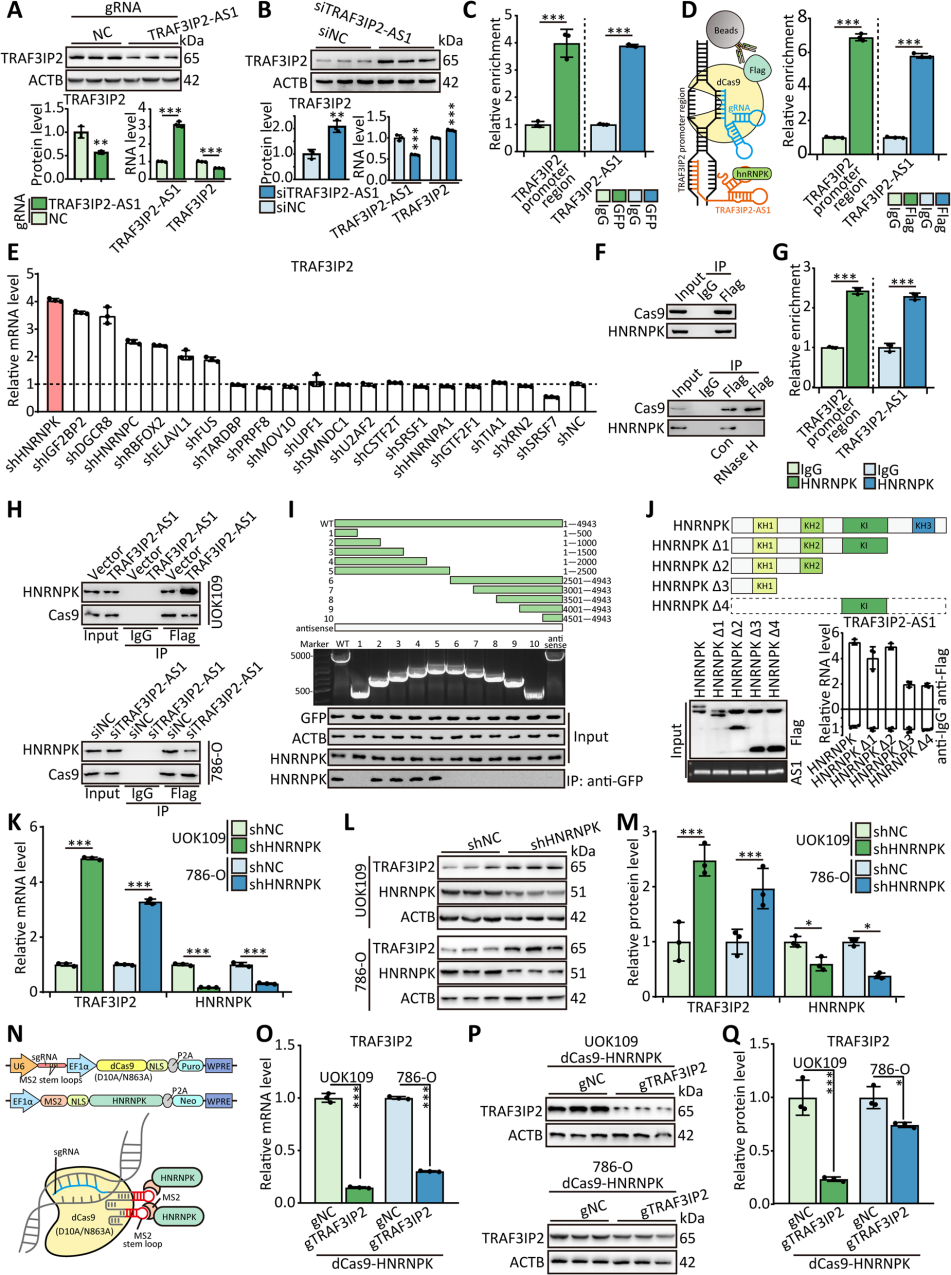
TRAF3IP2-AS1 down-regulates TRAF3IP2 by recruiting HNRNPK to TRAF3IP2 promoter. **A-B** The mRNA and protein levels of TRAF3IP2 were detected in UOK109 cells transfected with dCas9 and guide RNA targeting TRAF3IP2-AS1 promoter (gTRAF3IP2-AS1) and 786-O cells transfected with siRNA and antisense oligonucleotides (siTRAF3IP2-AS1). **C** UOK109 cells were transfected with MS2-GFP and TRAF3IP2-AS1-12 × MS2, then DNA region binding to TRAF3IP2-AS1 was enriched by anti-GFP antibody. **D** UOK109 cells were transfected with dCas9-Flag and gTRAF3IP2, then dCas9-ChIP assay was performed with anti-Flag antibody. **E** The mRNA level of TRAF3IP2 was detected by qRT-PCR after transfected with shRNA targeted the potential TRAF3IP2-AS1 binding proteins. **F** UOK109 cells were transfected with dCas9-Flag and gTRAF3IP2, then the proteins binding to the promoter region of *TRAF3IP2* was enriched by anti-Flag antibody. **G** UOK109 cells were lysed, then ChIP and RIP were performed with anti-HNRNPK antibody. **H** UOK109 cells and 786-O cells were transfected with dCas9-Flag, gTRAF3IP2 and indicated lentivirus, then the proteins binding to the promoter region of *TRAF3IP2* was enriched by anti-Flag antibody. **I** HEK293T cells were co-transfected with TRAF3IP2-AS1 truncations and MS2-GFP, then MS2-RIP assay was performed. **J** HEK293T cells were transfected with HNRNPK truncations, then RIP assay was performed. **K-M** The mRNA and protein levels of TRAF3IP2 and HNRNPK were detected in UOK109 cells and 786-O cells transfected shHNRNPK. **N** Schematic illustration of dCas9-based system to deliver the exogenous expressed protein to the promoter of *TRAF3IP2*. **O-Q** The mRNA and protein levels of TRAF3IP2 were detected in UOK109 cells and 786-O cells transfected dCas9-HNRNPK and gTRAF3IP2. The data are presented as the mean ± SD, *P < 0.05, **P < 0.01, ***P < 0.001

According to the previous report [28], lncRNA TRAF3IP2-AS1 suppresses the expression of TRAF3IP2 [12]. Overexpression of endogenous TRAF3IP2-AS1 by transfected with CRISPR-SAM and gRNA targeted *TRAF3IP2-AS1* promoter region (gTRAF3IP2-AS1) downregulated the protein and mRNA level of TRAF3IP2 in UOK109 cells (Fig. 4A), and the protein and mRNA level of TRAF3IP2 were increased in TRAF3IP2-AS1-silenced 786-O cells (Fig. 4B). However, the result of the MS2-RIP assay indicated that TRAF3IP2-AS1 could not directly bind to TRAF3IP2 mRNA (Figure S3). Interestingly, the result of the MS2-RIP assay showed that TRAF3IP2-AS1 and MS2-GFP complex enriched the *TRAF3IP2* promoter region (Fig. 4C), and the *TRAF3IP2* promoter region could be enriched by TRAF3IP2-AS1 probe according to the result of chromatin isolation by RNA purifications (CHIRP, Figure S4A). Similarly, the dCas9-ChIP assay showed the same phenomenon, suggesting that TRAF3IP2-AS1 might bind directly to the TRAF3IP2 promoter region (Fig. 4D). Overexpression of TRAF3IP2-AS1 enhanced the enrichment of TRAF3IP2-AS1 in the *TRAF3IP2* promoter region in UOK109 cells, and the reduced enrichment was observed in TRAF3IP2-AS1-slicenced 786-O cells (Figure S4D and S4E). Correspondingly, TRAF3IP2-AS1 with the mutation of the binding site could not bind to the *TRAF3IP2* promoter region, and overexpression of TRAF3IP2-AS1 with the mutation could not affect the expression of TRAF3IP2 (Figure S4F-G).

To uncover specific underlying mechanisms, UOK109 cells were transfected with negative control (shNC) or other silencing lentiviruses to knock down the top 20 interacting proteins of TRAF3IP2-AS1, and the result showed that the mRNA level of TRAF3IP2 was increased remarkably after transfection with shRNA targeting HNRNPK (shHNRNPK, Figs. 4E and S4B). Meanwhile, the result of dCas9-ChIP and RIP assays showed that HNRNPK was enriched in the *TRAF3IP2* promoter region (Fig. 4F-G), but the treatment of RNase H1 abolished the enrichment of HNRNPK in the *TRAF3IP2* promoter region (Figure S4C). Overexpression of TRAF3IP2-AS1 increased the recruitment of HNRNPK to the *TRAF3IP2* promoter region in UOK109 cells (Fig. 4H), and downregulation of TRAF3IP2-AS1 attenuated HNRNPK binding to *TRAF3IP2* promoter in 786-O cells. These results indicated that HNRNPK could bind to the *TRAF3IP2* promoter region through interacting with TRAF3IP2-AS1. To further characterize the binding motif, ten truncated TRAF3IP2-AS1 plasmids were transfected into HEK 293 T cells. The 1–1000 nt fragment of TRAF3IP2-AS1 was sufficient to bind HNRNPK, but not 1–500 nt, suggesting that the binding site was between 500–1000 nt (Fig. 4I). In parallel, we generated HNRNPK truncations to perform a RIP assay, and the data indicated that the KH1 domain of HNRNPK is critical for binding to TRAF3IP2-AS1 (Fig. 4J).

The protein and mRNA levels of TRAF3IP2 were increased markedly after transfection with shHNRNPK in UOK109 and 786-O cells (Fig. 4K-M). To delve further into one potential function of the HNRNPK in tumor progression of *NONO-TFE3* tRCC, we constructed a dCas9-based system to recruit HNRNPK to the *TRAF3IP2* promoter by MS2-loop on gRNA. The data of western blot and qPCR showed that the protein and mRNA levels of TRAF3IP2 were decreased clearly in UOK109 and 786-O cells transfected with gRNA targeted to the *TRAF3IP2* promoter (gTRAF3IP2, Fig. 4N-Q). In order to determine the impact of HNRNPK on UOK109 and 786-O behavior, the following assays were performed: colony formation, tumor sphere formation, EdU, flow cytometry, and Transwell assays. Transfection of UOK109 cells with dCas9-HNRNPK and gTRAF3IP2 inhibited cell proliferation, cell cycle, migration, and invasion compared to the negative control (Figure S5A-G and S5I-K). Studies using flow cytometry revealed that dCas9-HNRNPK and gTRAF3IP2 significantly increased apoptosis in UOK109 (Figure S5H). Taken together, these results indicate that TRAF3IP2-AS1 mediates *NONO-TFE3* tRCC progression by recruiting HNRNPK to the promoter region of *TRAF3IP2* to suppress the expression of TRAF3IP2.

### HNRNPK suppresses TRAF3IP2 via DNA methylation by recruiting DNMT1

**Fig. 5:**
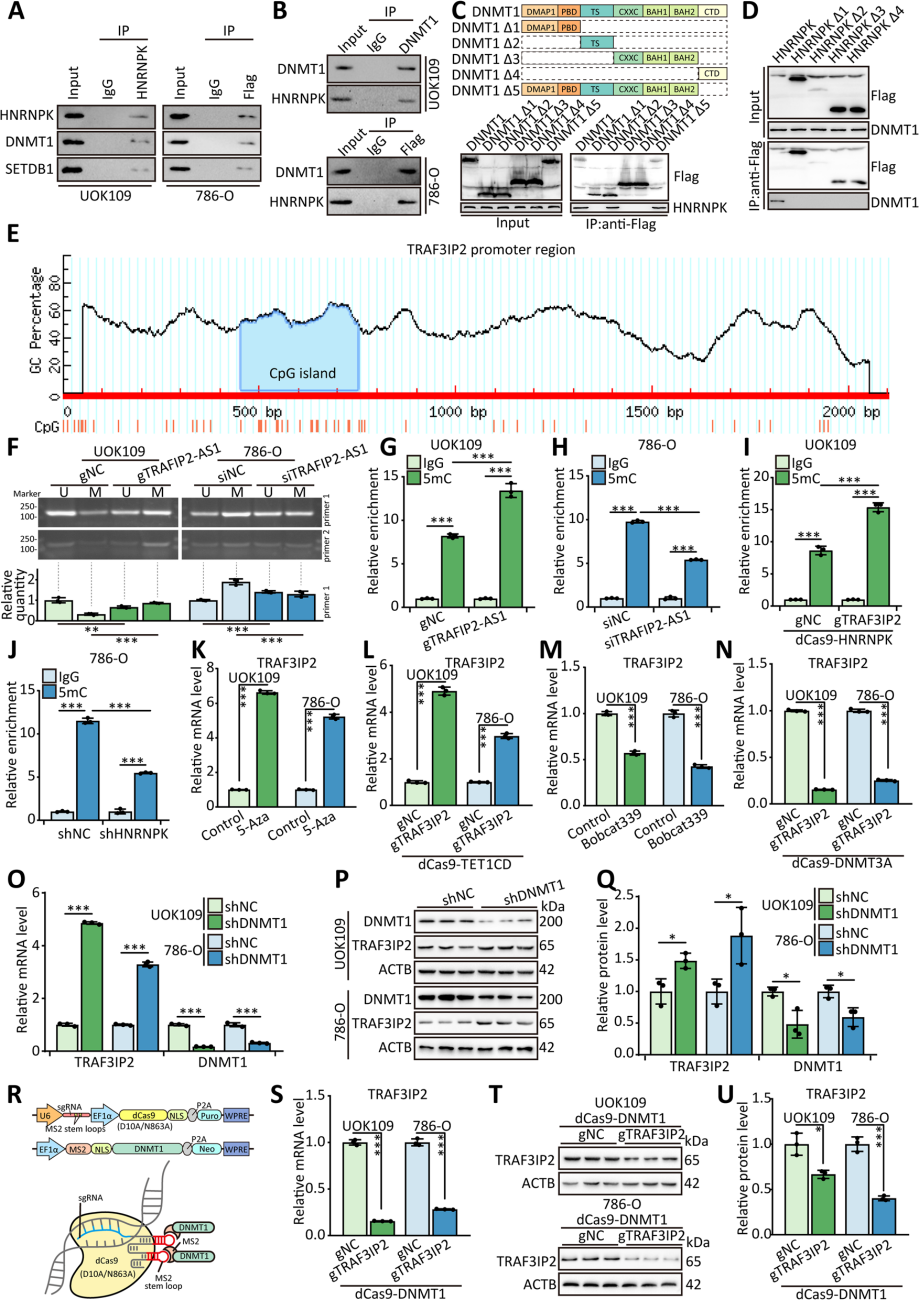
HNRNPK suppresses TRAF3IP2 via DNA methylation via recruiting DNMT1. **A-B** 786-O cells transfected with Flag-HNRNPK/Flag-DNMT1 and UOK109 cells were lysed, then CoIP assay was performed with anti-HNRNPK antibody, anti-Flag antibody, anti-DNMT1 antibody or normal rabbit IgG. **C-D** HEK 293 T cells were transfected with DNMT1 truncations or HNRNPK truncations, then CoIP assay was performed. **E** The CpG island located in the promoter region of *TRAF3IP2* was predicted by MethPrimer. **F** Bisulfite PCR of CpG island located in the promoter region of *TRAF3IP2* following bisulfite conversion in UOK109 cells and 786-O cells transfected with indicated lentivirus. **G-J** UOK109 cells and 786-O cells were transfected with indicated lentivirus, then MeDIP assay was performed with anti-5mC antibody. **K-N** UOK109 cells and 786-O cells were transfected with indicated lentivirus or treatment with indicated inhibitors, then the mRNA level of TRAF3IP2 was analyzed. **O-Q** The mRNA and protein levels of TRAF3IP2 and DNMT1 were detected in UOK109 cells and 786-O cells transfected shDNMT1. **R** Schematic illustration of dCas9-based system to deliver the exogenous expressed protein to the promoter of *TRAF3IP2*. **S-U** The mRNA and protein levels of TRAF3IP2 were detected in UOK109 cells and 786-O cells transfected dCas9-DNMT1 and gTRAF3IP2. The data are presented as the mean ± SD, *P < 0.05, **P < 0.01, ***P < 0.001

Considering that HNRNPK binds to DNMT1 and SETDB1 in mouse embryonic fibroblasts [28], we confirmed the interaction between HNRNPK and DNMT1/SETDB1 in UOK109 and 786-O cells by CoIP assays (Fig. 5A and 5B). Furthermore, CoIP assays using truncated DNMT1 demonstrated that the TS domain of DNMT1 interacted with HNRNPK (Fig. 5C), and CoIP assays using truncated HNRNPK indicated that DNMT1 could not interact with HNRNPK lacking the KH3 domain (Fig. 5D). According to the biological function of DNMT1, we analyzed the *TRAF3IP2* promoter region and found a complete CpG island by MethPrimer online software [29] (www.urogene.org/cgi-bin/methprimer/methprimer.cgi, Fig. 5E). To explore the influence of TRAF3IP2-AS1 on the *TRAF3IP2* promoter, primers specific for methylated (M) or unmethylated (U) CpG sites were designed to amplify the genomic DNA after bisulfite modification. The result indicated that overexpression of TRAF3IP2-AS1 increased the level of DNA methylation on the *TRAF3IP2* promoter region in UOK109 cells (Fig. 5F). Likewise, the level of DNA methylation was decreased in TRAF3IP2-AS1-silenced 786-O cells. The same result was obtained in the MeDIP assay (Fig. 5G and H). As expected, the level of DNA methylation was increased in UOK109 cells transfected with dCas9-HNRNPK and gTRAF3IP2 and decreased in 786-O cells with shHNRNPK (Fig. 5I-J).

To visualize the effect of DNA methylation on the expression of TRAF3IP2, we treated the cells with the DNA demethylated agent 5-aza-2’-deoxycytidine (5-Aza) or gTRAF3IP2 combined with dCas9-TET1CD, which construct an epigenome editing effector to remove DNA methylation. The result of qPCR indicated that the mRNA level of TRAF3IP2 increased remarkably after removing DNA methylation (Fig. 5K and L). Meanwhile, the mRNA level of TRAF3IP2 was decreased markedly in UOK109 and 786-O cells treated with Bobcat339, a novel cytosine-based TET enzyme inhibitor, or transfected gTRAF3IP2 combined with dCas9-DNMT3A, which could manipulate DNA methylation (Fig. 5M-N). The protein and mRNA levels of TRAF3IP2 were increased markedly after transfection with shDNMT1 in UOK109 and 786-O cells (Fig. 5O-Q). A dCas9-based system to recruit DNMT1 to the *TRAF3IP2* promoter was constructed to clarify the function of the DNMT1 in the development of *NONO-TFE3* tRCC. The data of western blot and qPCR showed that the protein and mRNA levels of TRAF3IP2 were suppressed clearly in UOK109 and 786-O cells transfected with gTRAF3IP2 (Fig. 5R-U).

To further illuminate the biological function of DNMT1 in *NONO-TFE3* tRCC, gTRAF3IP2 combined with dCas9-DNMT1 was used to upregulate DNMT1 expression in UOK109. In CCK-8 assays, gTRAF3IP2 and dCas9-DNMT1 clearly inhibited the proliferation of UOK109 cells (Figure S6A). Furthermore, the results of colony formation, tumor sphere formation, EdU, flow cytometry, and Transwell assays revealed that cell proliferation, cell cycle, migration, and invasion were enhanced by transfection with gTRAF3IP2 combined with dCas9-DNMT1 compared with the negative control in UOK109 cells (Figure S6B-I, S6L-M). Flow cytometric experiments showed that increased DNMT1 expression obviously inhibited apoptosis in UOK109 cells (Figure S6J and S6K). Collectively, the results reveal that TRAF3IP2-AS1 functions as a scaffold to recruit HNRNPK and DNMT1 to the *TRAF3IP2* promoter region.

### HNRNPK suppresses TRAF3IP2 via H3K9me3 through recruiting SETDB1

**Fig. 6:**
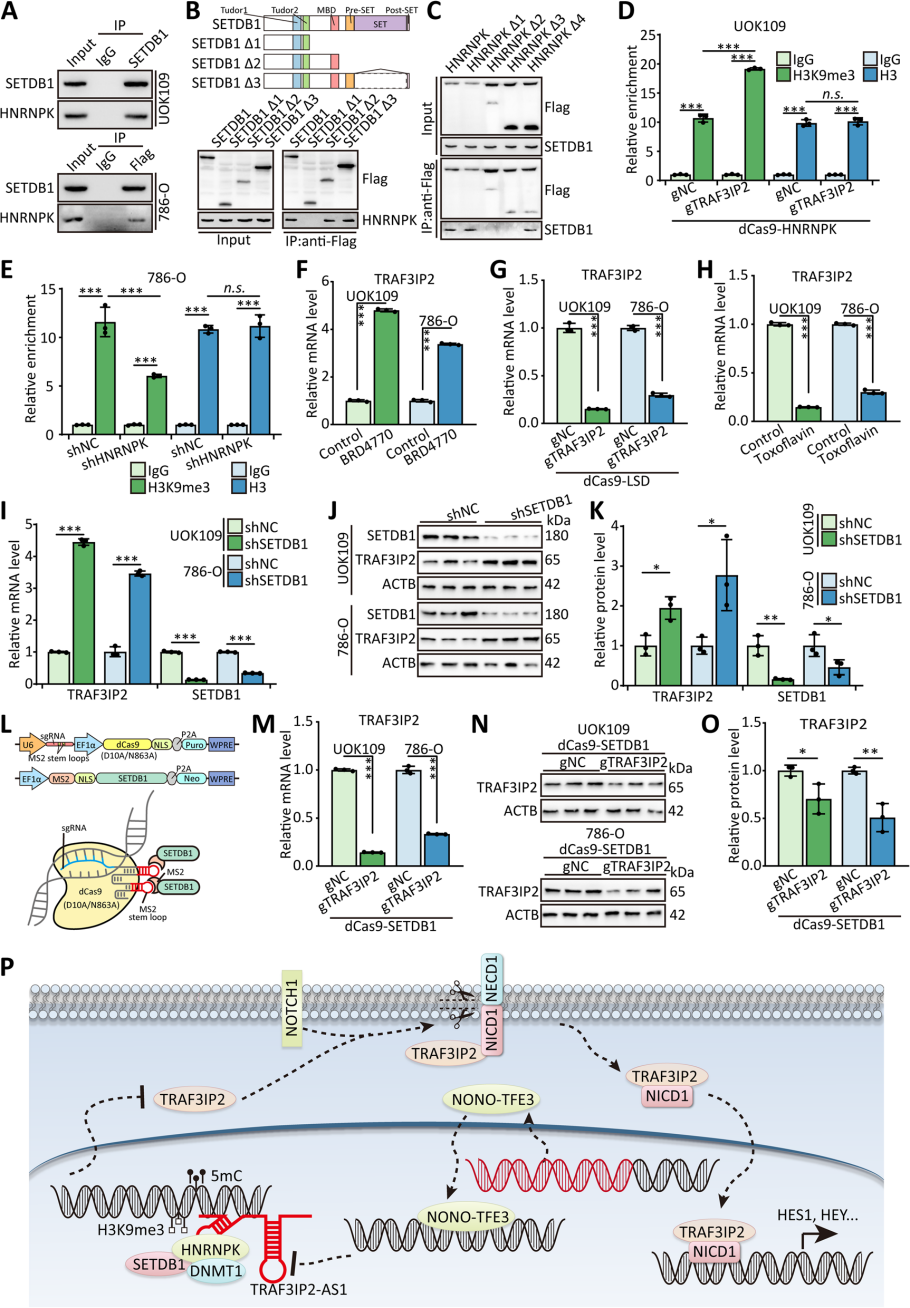
HNRNPK suppresses TRAF3IP2 via H3K9me3 through recruiting SETDB1. **A** 786-O cells transfected with Flag-SETDB1 and UOK109 cells were lysed, then CoIP assay was performed with anti-SETDB1 antibody, anti-Flag antibody or normal rabbit IgG. **B-C** HEK 293 T cells were transfected with SETDB1 truncations or HNRNPK truncations, then CoIP assay was performed. **D-E** UOK109 cells and 786-O cells were transfected with indicated lentivirus, then ChIP assay was performed with anti-H3 antibody or anti- H3K9me3 antibody. **F–H** UOK109 cells and 786-O cells were transfected with indicated lentivirus or treatment with indicated inhibitors, then the mRNA level of TRAF3IP2 was analyzed. **I-K** The mRNA and protein levels of TRAF3IP2 and SETDB1 were detected in UOK109 cells and 786-O cells transfected shSETDB1. **L** Schematic illustration of dCas9-based system to deliver the exogenous expressed protein to the promoter of *TRAF3IP2*. **M–O** The mRNA and protein levels of TRAF3IP2 were detected in UOK109 cells and 786-O cells transfected dCas9-SETDB1 and gTRAF3IP2. **P** Schematic diagram for the mechanisms of that TRAF3IP2 and NONO-TFE3 fusion accelerate tumor progression of NONO-TFE3 tRCC and that lncRNA TRAF3IP2-AS1 recruits HNRNPK, DNMT1 and SETDB1 to the promoter region of TRAF3IP2 and represses the expression of TRAF3IP2 via mediating 5mC on DNA and H3K9me3. The data are presented as the mean ± SD, *P < 0.05, **P < 0.01, ***P < 0.001

According to the previous analysis [28], the interaction between HNRNPK and SETDB1 was confirmed by CoIP assays (Fig. 6A). The result of CoIP assays using truncated SETDB1 demonstrated that the MBD domain of SETDB1 interacted with HNRNPK (Fig. 6B). In parallel, we generated HNRNPK truncations to perform the CoIP assay, and the data indicated that the KI domain of HNRNPK is critical for binding to SETDB1 (Fig. 6C). Depending on the biological function of SETDB1, we performed the ChIP assay using the H3K9me3 antibody or H3 antibody. The result showed that the enrichment of the *TRAF3IP2* promoter region was significantly accumulated after transfection with gTRAF3IP2 and dCas9-HNRNPK in the H3K9me3-ChIP assay (Fig. 6D-E), but not H3-ChIP, while the silence of HNRNPK led to inverse effects in 786-O cells.

To assess the effect of histone methylation on the expression of TRAF3IP2, we treated the cells with BRD4770, a histone methyltransferase G9a inhibitor. The result of qPCR indicated that the mRNA level of TRAF3IP2 increased remarkably after removing histone methylation (Fig. 6F). Meanwhile, the mRNA level of TRAF3IP2 was decreased markedly in UOK109 and 786-O cells treated with Toxoflavin, an inhibitor of KDM4A, or transfected gTRAF3IP2 combined with dCas9-LSD1, which could manipulate lysine-specific demethylation of histone (Fig. 6G and H). The protein and mRNA levels of TRAF3IP2 were increased markedly after transfection with shSETDB1 in UOK109 and 786-O cells (Fig. 6I-K). A dCas9-based system designed to recruit SETDB1 to the *TRAF3IP2* promoter was constructed to clarify the function of the SETDB1 in the progression of *NONO-TFE3* tRCC. The data of western blot and qPCR showed that the protein and mRNA levels of TRAF3IP2 were suppressed clearly in UOK109 and 786-O cells transfected with gTRAF3IP2 (Fig. 6L-O).

To further explore the biological function of SETDB1 in *NONO-TFE3* tRCC, CCK-8, colony formation, tumor sphere formation, EdU, flow cytometry, and Transwell assays were performed. The results revealed that cell proliferation, cell cycle, migration, and invasion were inhibited by gTRAF3IP2 combined with dCas9-SETDB1 compared with the negative control in UOK109 cells (Figure S7A-I and S7L-M). Flow cytometric experiments showed that gTRAF3IP2 combined with dCas9-SETDB1 obviously increased the apoptosis of UOK109 cells (Figure S7J and S7K). Overall, HNRNPK could modulate the progression of *NONO-TFE3* tRCC by recruiting SETDB1 to the promoter region of *TRAF3IP2*.

## Discussion

Apart from acting as ceRNA or interacting with mRNAs or RNA binding proteins, increasing studies revealed that lncRNAs are also shown to regulate the transcription of target genes via binding to the promoter region [26, 27]. Here, we demonstrated that lncRNA TRAF3IP2-AS1 bound to the promoter region of *TRAF3IP2* and inhibited the expression of TRAF3IP2. Meanwhile, we uncovered that HNRNPK could bind to lncRNA TRAF3IP2-AS1 and be recruited to the promoter region of *TRAF3IP2* and inhibit the expression of TRAF3IP2.

Typically, HNRNPs exert their function through interacting with other proteins, leading to different outcomes of tumor progression. HNRNPA1/2 proteins may interact with NEK2 to regulate PKM splicing and promote aerobic glycolysis in multiple myeloma [30]. KHSRP (KH-type splicing regulatory protein) could promote invasion and metastasis of non-small-cell lung cancer by interacting with HNRNPC [31]. Surprisingly, PSTAR (p53-stabilizing and activating RNA) could interact with HNRNPK and enhance the SUMOylation of HNRNPK. Subsequently, HNRNPK serves as a co-activator of p53 to arrest the cell cycle of hepatocellular carcinoma [32]. Here, we revealed that HNRNPK could interact with DNMT1 and SETDB1, respectively, and HNRNPK/DNMT1/SETDB1 complex might be recruited to the promoter region of *TRAF3IP2* by the guidance of TRAF3IP2-AS1.

Epigenetic modifications, such as DNA methylation and histone modifications, have been proposed to play essential roles in maintaining the stemness of tumor cells [33, 34]. Although the study of Chen indicated that global 5mC levels do not significantly change during kidney tumorigenesis [35], more reliable evidence suggests that the distribution of 5mC in the gene body was altered during the tumor initiation and development [36, 37]. Generally, the 5mC located in the promoter region of genes inhibits the initiation of transcription. In many tumor cells, the lower level of 5mC was observed at the promoter of oncogenes [38, 39]. 5mC on DNA would induce the histone modifications, such as H3K9me3, and tightly pack nucleosomes to further lock the expression of genes [40, 41]. We found that DNMT1 and SETDB1 recruited by HNRNPK could modify the DNA methylation and histone modifications of the *TRAF3IP2* promoter region, respectively, leading to downregulation of TRAF3IP2.

Although the NOTCH1 pathway is not fully understood, it is common sense that the NOTCH1 pathway is involved in the development of various cancers [42, 43]. In non-small-cell lung cancer, high expression of NOTCH1 enhances tumor proliferation and lymphatic metastasis [44]. NOTCH1 and oncogenic gene Ras synergistically facilitate the epithelial-mesenchymal transition in pancreatic cancer [45]. In the glioblastoma hypoxic microenvironment, the NOTCH1 pathway is activated by the target gene of hypoxia-inducible factor for the adaptation to low oxygen of tumor cells [46]. In this study, our data indicated that TRAF3IP2 could function as a co-activator of NOTCH1 and activate the NOTCH1 pathway to promote the progression of *NONO-TFE3* tRCC.

In summary, our study uncovered a crucial role of TRAF3IP2 in the development of *NONO-TFE3* tRCC. High expression of TRAF3IP2 was caused by the low expression of TRAF3IP2-AS1 via DNA/Histone modification and promoted the development of *NONO-TFE3* tRCC (Fig. 6P).

## Supplementary Information

**Additionalfile 1: Figure S1.** (A) The protein level of TRAF3IP2 after up-/down-regulated TRAF3IP2. (B) The effects of TRAF3IP2 knockdown on the proliferation of UOK109 and 786-O cells respectively were examined by CCK-8 assay. The data are presented as the mean ± SD, **P<0.01, ***P< 0.001. **Figure S2**. NOTCH1 pathway induces development of *NONO-TFE3* tRCC. (A-E) The effects of NICD1 overexpression or NOTCH1 knockdown on the proliferation of  UOK109 and 786-O cells respectively were examined by CCK-8 assay (A-B), colony formation assays (C-D) and tumor sphere formation (E). (F) EdU assays were used to detect the proliferationrate of  UOK109 and 786-O cells after transfection for 48h. (G) Cell cycle was analyzed using flow cytometry after transfection for 48h. H Cell apoptosis was analyzed via flow cytometry using an Annexin V/PI kit after transfection for 48h. (I-K) Migration and invasion assays were performed with transfected cells using Transwell inserts. The data are presented as the mean ± SD, *P< 0.05, **P< 0.01, ***P<0.001. **FigureS3.** Level of TRAF3IP2 mRNA detected by qRT-PCRafter MS2-RIP for GFP in UOK109 cells. AS1, NC and AS1-antisense correspond to TRAF3IP2-AS1, empty vector and TRAF3IP2-AS1-antisense. The data are presented as the mean ± SD. **Figure S4.** TRAF3IP2-AS1 down-regulates TRAF3IP2 by recruiting HNRNPK to TRAF3IP2 promoter. (A) UOK109 cells were lysed, then CHIRP were performed with TRAF3IP2-AS1 probe or LacZ probe. (B) The mRNA levels were detected by qRT-PCR after transfected with shRNA targeted the potential TRAF3IP2-AS1 binding proteins. (C) UOK109 cells were lysed, then ChIP were performed with anti-HNRNPK antibody. (D-E) UOK109 cells were transfected with indicated lentivirus and siRNAs, then the promoter region of TRAF3IP2 and TRAF3IP2-AS1 were enriched by TRAF3IP2-AS1 probe. (F) UOK109 cells were co-transfected with TRAF3IP2-AS1/ TRAF3IP2-AS1^Mut^ and MS2-GFP, then MS2-RIP assay was performed. (G) The mRNA level of TRAF3IP2 were detected in UOK109 cellstransfected TRAF3IP2-AS1/TRAF3IP2-AS1^Mut^. The data are presented as the mean ± SD, **P< 0.01, ***P< 0.001. **FigureS5.** HNRNPK inhibits development of *NONO-TFE3* tRCC by down-regulating the expression of TRAF3IP2. (A-E) The effects of HNRNPK on the proliferation of UOK109 and 786-O cells respectively were examined by CCK-8 assay (A-B), colony formation assays (C-D) and tumor sphere formation (E). (F) EdU assays were used to detect the proliferation rate of UOK109 and 786-O cells after transfection for 48h. (G) Cellcycle was analyzed using flow cytometry after transfection for 48h. (H) Cellapoptosis was analyzed via flow cytometry using an Annexin V/PI kit after transfection for 48h. (I-K) Migration and invasion assays were performed with transfected cells using Transwell inserts. The data are presented as the mean ± SD, **P< 0.01, ***P< 0.001. **Figure S6.** DNMT1 inhibits development of *NONO-TFE3* tRCC by down-regulating the expression of TRAF3IP2. (A-E) The effects of DNMT1 on the proliferation of UOK109 cells respectively were examined by CCK-8 assay (A), colony formation assays (B-C) and tumor sphere formation (D-E). (F-G) EdU assays were used to detect the proliferation rate of UOK109 cells after transfection for 48h. (H-I) Cell cycle was analyzed using flow cytometry after transfection for 48h. (J-K) Cell apoptosis was analyzed via flow cytometry using an Annexin V/PI kit after transfection for 48h. (L-M) Migration and invasion assays were performed with transfected cells using Transwell inserts.The data are presented as the mean ± SD, *P< 0.05, **P< 0.01, ***P<0.001. **TableS1. **Primers used for real-time PCR. **Table S2. **Primers used for ChIP/CHIRP/MS2-RIP assay. **Table S3.** Primary antibodies used in this study. **Table S4. **Primers used for MSP analysis. **Table S5. **Small-molecule inhibitors used in this study. **Table S6.** SiRNA, shRNA and ASOs used for silencing target genes. **Table S7.** Guide RNA used for CRISPR/dCas9 system. **Table S8.** Primers used for MSP analysis.

## Data Availability

The datasets used and/or analyzed during the current study are available from the corresponding author on reasonable request.

## Abbreviations

ChIP: Chromatin immunoprecipitation
CHIRP: Chromatin Isolation by RNA Purification
CoIP: Co-Immunoprecipitation
CRISPR: Clustered regularly interspaced short palindromic repeats
DNMT1: DNA methyltransferase 1
GFP: Green fluorescent protein
gRNA: Guide RNA
H3K9me3: Trimethylated lysine 9 of histone H3
HNRNPK: Heterogeneous nuclear ribonucleoprotein K
m^6^A: *N*^6^-Methyladenosine
MeDIP: Methylated DNA Immunoprecipitation
MiT/TFE: Microphthalmia family of bHLH-LZ transcription factors
NONO: Non-POU domain containing octamer binding
PRCC: Proline rich mitotic checkpoint control factor
RIP: RNA immunoprecipitation
SETDB1: SET domain bifurcated histone lysine methyltransferase 1
TFE3: Transcription factor binding to IGHM enhancer 3
TRAF3IP2: TRAF3 interacting protein 2
TRAF3IP2-AS1: TRAF3IP2 antisense RNA 1
tRCC: Translocation renal cell carcinoma
