# Supplementary material for: 5mC and H3K9me3 of TRAF3IP2 promoter region accelerates the progression of translocation renal cell carcinoma
Source: Biomark Res. 2022 Jul 27;10:54. doi: 10.1186/s40364-022-00402-3 (PMC9331078; doi:10.1186/s40364-022-00402-3)
Supplement: Supplementary file 1 — Additionalfile 1: Figure S1. (A) The protein level of TRAF3IP2 after up-/down-regulated TRAF3IP2. (B) The effects of TRAF3IP2 knockdown on the proliferation of UOK109 and 786-O cells respectively were examined by CCK-8 assay. The data are presented as the mean ± SD, **P<0.01, ***P< 0.001. Figure S2. NOTCH1 pathway induces development of NONO-TFE3 tRCC. (A-E) The effects of NICD1 overexpression or NOTCH1 knockdown on the proliferation of UOK109 and 786-O cells respectively were examined by CCK-8 assay (A-B), colony formation assays (C-D) and tumor sphere formation (E). (F) EdU assays were used to detect the proliferationrate of UOK109 and 786-O cells after transfection for 48h. (G) Cell cycle was analyzed using flow cytometry after transfection for 48h. H Cell apoptosis was analyzed via flow cytometry using an Annexin V/PI kit after transfection for 48h. (I-K) Migration and invasion assays were performed with transfected cells using Transwell inserts. The data are presented as the mean ± SD, *P< 0.05, **P< 0.01, ***P<0.001. FigureS3. Level of TRAF3IP2 mRNA detected by qRT-PCRafter MS2-RIP for GFP in UOK109 cells. AS1, NC and AS1-antisense correspond to TRAF3IP2-AS1, empty vector and TRAF3IP2-AS1-antisense. The data are presented as the mean ± SD. Figure S4. TRAF3IP2-AS1 down-regulates TRAF3IP2 by recruiting HNRNPK to TRAF3IP2 promoter. (A) UOK109 cells were lysed, then CHIRP were performed with TRAF3IP2-AS1 probe or LacZ probe. (B) The mRNA levels were detected by qRT-PCR after transfected with shRNA targeted the potential TRAF3IP2-AS1 binding proteins. (C) UOK109 cells were lysed, then ChIP were performed with anti-HNRNPK antibody. (D-E) UOK109 cells were transfected with indicated lentivirus and siRNAs, then the promoter region of TRAF3IP2 and TRAF3IP2-AS1 were enriched by TRAF3IP2-AS1 probe. (F) UOK109 cells were co-transfected with TRAF3IP2-AS1/ TRAF3IP2-AS1Mut and MS2-GFP, then MS2-RIP assay was performed. (G) The mRNA level of TRAF3IP2 were detected in UOK109 c [file 40364_2022_402_MOESM1_ESM.docx]

**Supplementary Figures**


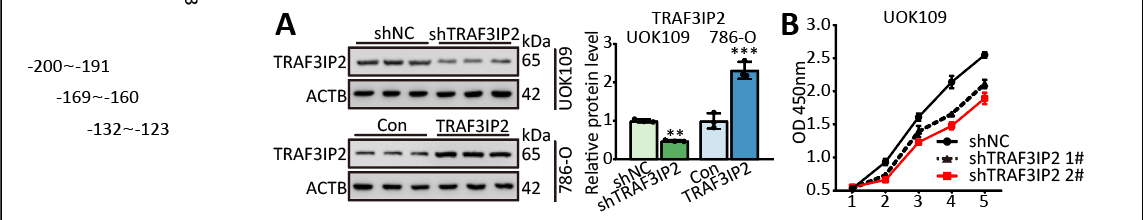


**Figure S1 (A)** The protein level of TRAF3IP2 after up-/down-regulated TRAF3IP2. **(B)** The effects of TRAF3IP2 knockdown on the proliferation of UOK109 and 786-O cells respectively were examined by CCK-8 assay.The data are presented as the mean ± SD, ***P*< 0.01, ****P*< 0.001


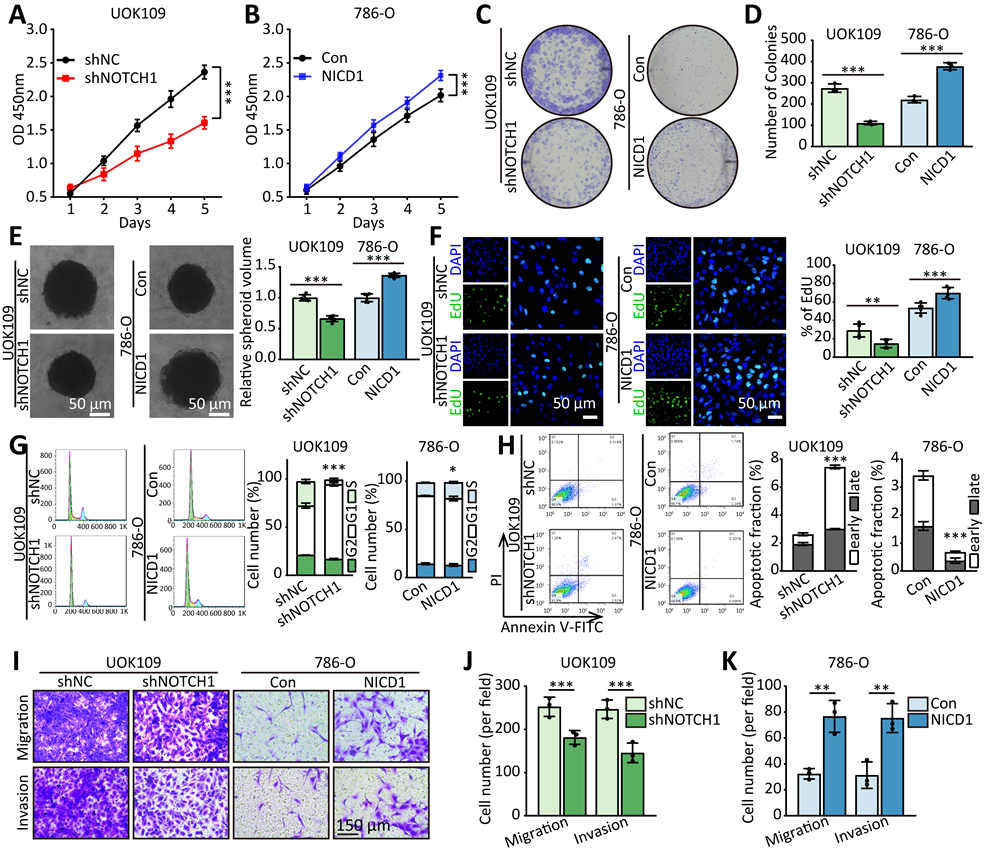


**Figure S2 NOTCH1 pathway induces development of *NONO-TFE3* tRCC. (A-E)** The effects of NICD1 overexpression or NOTCH1 knockdown on the proliferation of UOK109 and 786-O cells respectively were examined by CCK-8 assay **(A-B)**, colony formation assays **(C-D)** and tumor sphere formation **(E)**. **(F)** EdU assays were used to detect the proliferation rate of UOK109 and 786-O cells after transfection for 48h. **(G)** Cell cycle was analyzed using flow cytometry after transfection for 48h. **H** Cell apoptosis was analyzed via flow cytometry using an Annexin V/PI kit after transfection for 48h. **(I-K)** Migration and invasion assays were performed with transfected cells using Transwell inserts. The data are presented as the mean ± SD, **P*< 0.05, ***P*< 0.01, ****P*< 0.001

**
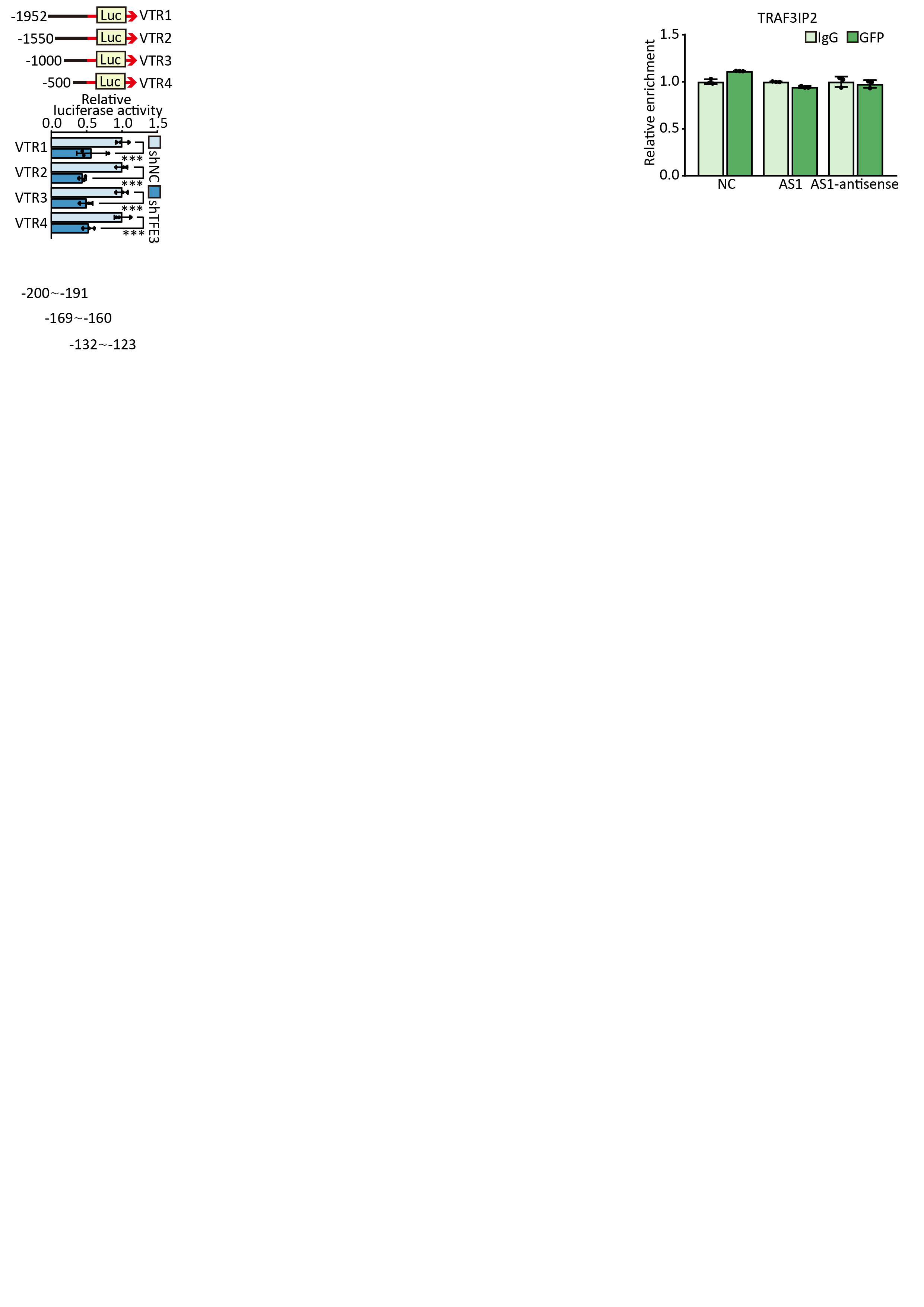
**

**Figure S3** Level of TRAF3IP2 mRNA detected by qRT-PCR after MS2-RIP for GFP in UOK109 cells. AS1, NC and AS1-antisense correspond to TRAF3IP2-AS1, empty vector and TRAF3IP2-AS1-antisense. The data are presented as the mean ± SD.

**
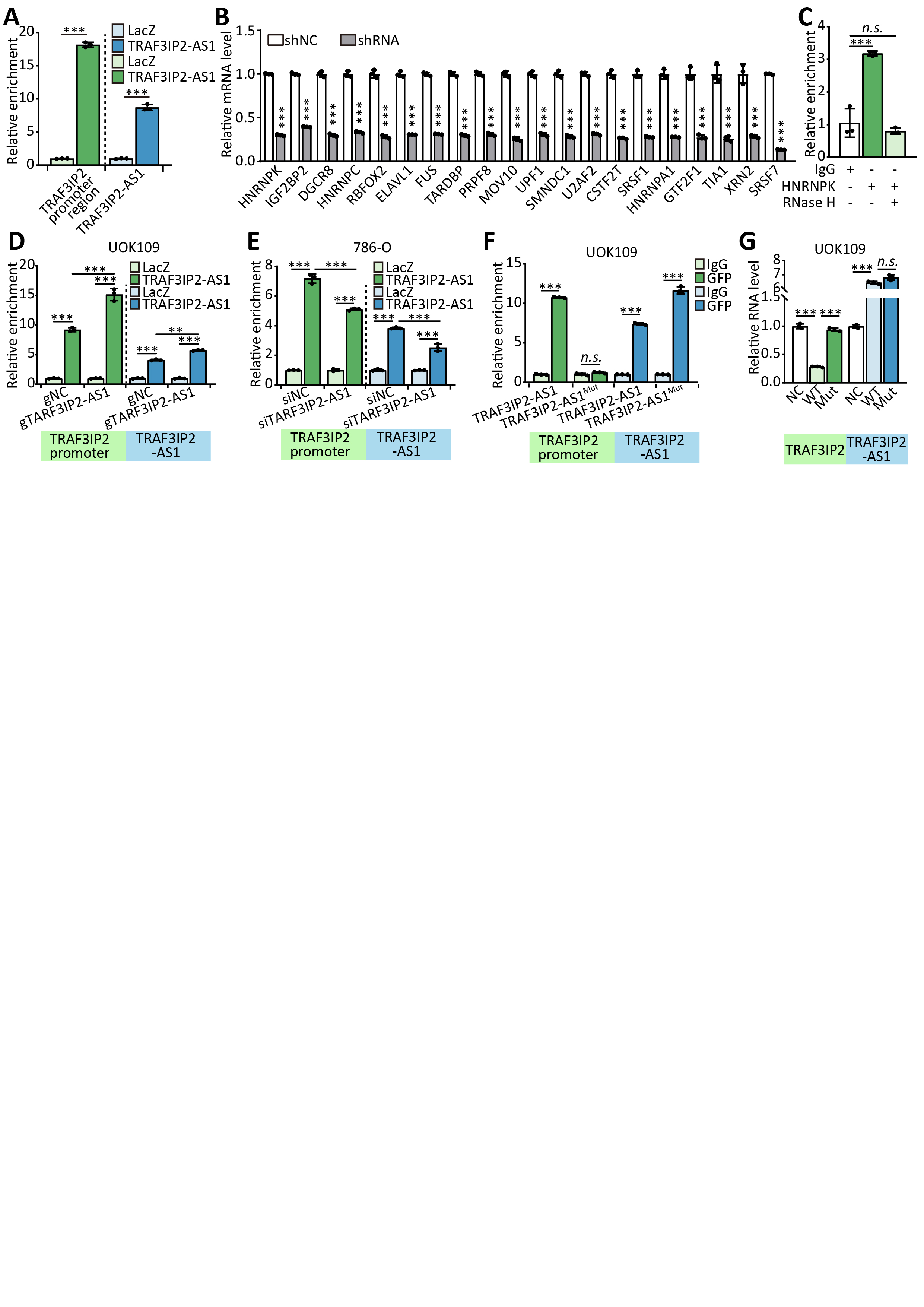
**

**Figure S4 TRAF3IP2-AS1 down-regulates TRAF3IP2 by recruiting HNRNPK to TRAF3IP2 promoter. (A)** UOK109 cells were lysed, then CHIRP were performed with TRAF3IP2-AS1 probe or LacZ probe. **(B)** The mRNA levels were detected by qRT-PCR after transfected with shRNA targeted the potential TRAF3IP2-AS1 binding proteins. **(C)** UOK109 cells were lysed, then ChIP were performed with anti-HNRNPK antibody. **(D-E)** UOK109 cells were transfected with indicated lentivirus and siRNAs, then the promoter region of TRAF3IP2 and TRAF3IP2-AS1 were enriched by TRAF3IP2-AS1 probe. **(F)** UOK109 cells were co-transfected with TRAF3IP2-AS1/ TRAF3IP2-AS1^Mut^ and MS2-GFP, then MS2-RIP assay was performed. **(G)** The mRNA level of TRAF3IP2 were detected in UOK109 cells transfected TRAF3IP2-AS1/TRAF3IP2-AS1^Mut^. The data are presented as the mean ± SD, ***P*< 0.01, ****P*< 0.001


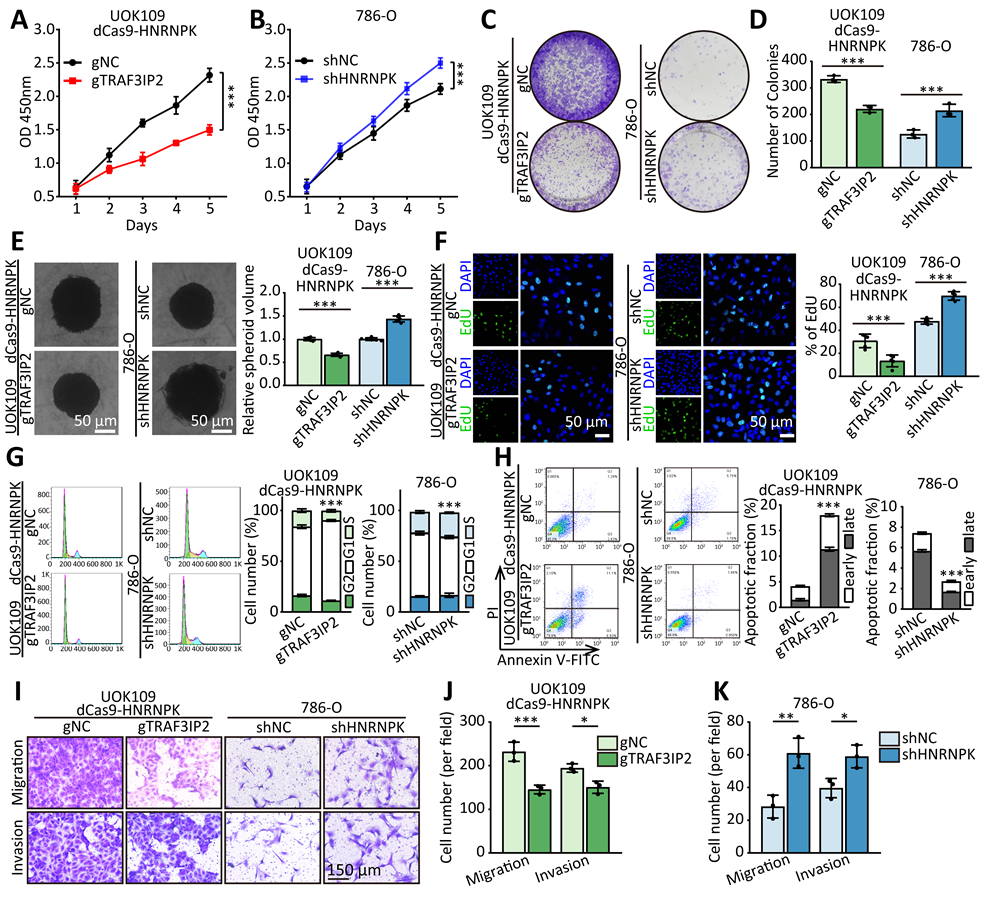


**Figure S5 HNRNPK inhibits development of *NONO-TFE3* tRCC by down-regulating the expression of TRAF3IP2. (A-E)** The effects of HNRNPK on the proliferation of UOK109 and 786-O cells respectively were examined by CCK-8 assay **(A-B)**, colony formation assays **(C-D)** and tumor sphere formation **(E)**. **(F)** EdU assays were used to detect the proliferation rate of UOK109 and 786-O cells after transfection for 48h. **(G)** Cell cycle was analyzed using flow cytometry after transfection for 48h. **(H)** Cell apoptosis was analyzed via flow cytometry using an Annexin V/PI kit after transfection for 48h. **(I-K)** Migration and invasion assays were performed with transfected cells using Transwell inserts. The data are presented as the mean ± SD, ***P*< 0.01, ****P*< 0.001


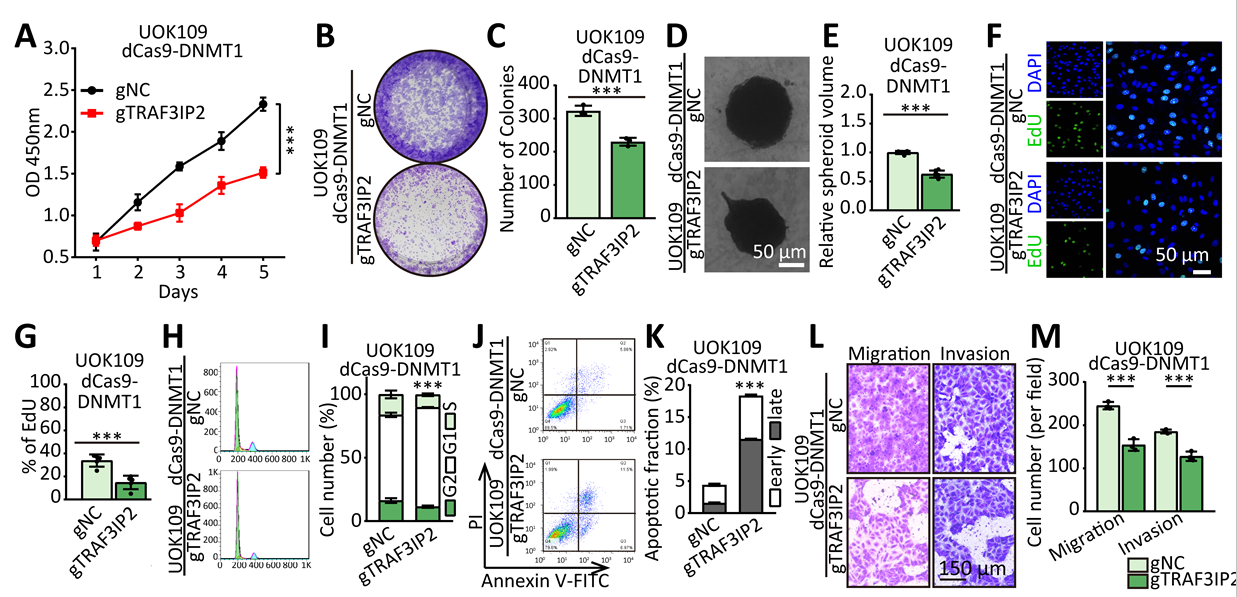


**Figure S6 DNMT1 inhibits development of *NONO-TFE3* tRCC by down-regulating the expression of TRAF3IP2. (A-E)** The effects of DNMT1 on the proliferation of UOK109 cells respectively were examined by CCK-8 assay **(A)**, colony formation assays **(B-C)** and tumor sphere formation **(D-E)**. **(F-G)** EdU assays were used to detect the proliferation rate of UOK109 cells after transfection for 48h. **(H-I)** Cell cycle was analyzed using flow cytometry after transfection for 48h. **(J-K)** Cell apoptosis was analyzed via flow cytometry using an Annexin V/PI kit after transfection for 48h. **(L-M)** Migration and invasion assays were performed with transfected cells using Transwell inserts. The data are presented as the mean ± SD, **P*< 0.05, ***P*< 0.01, ****P*< 0.001


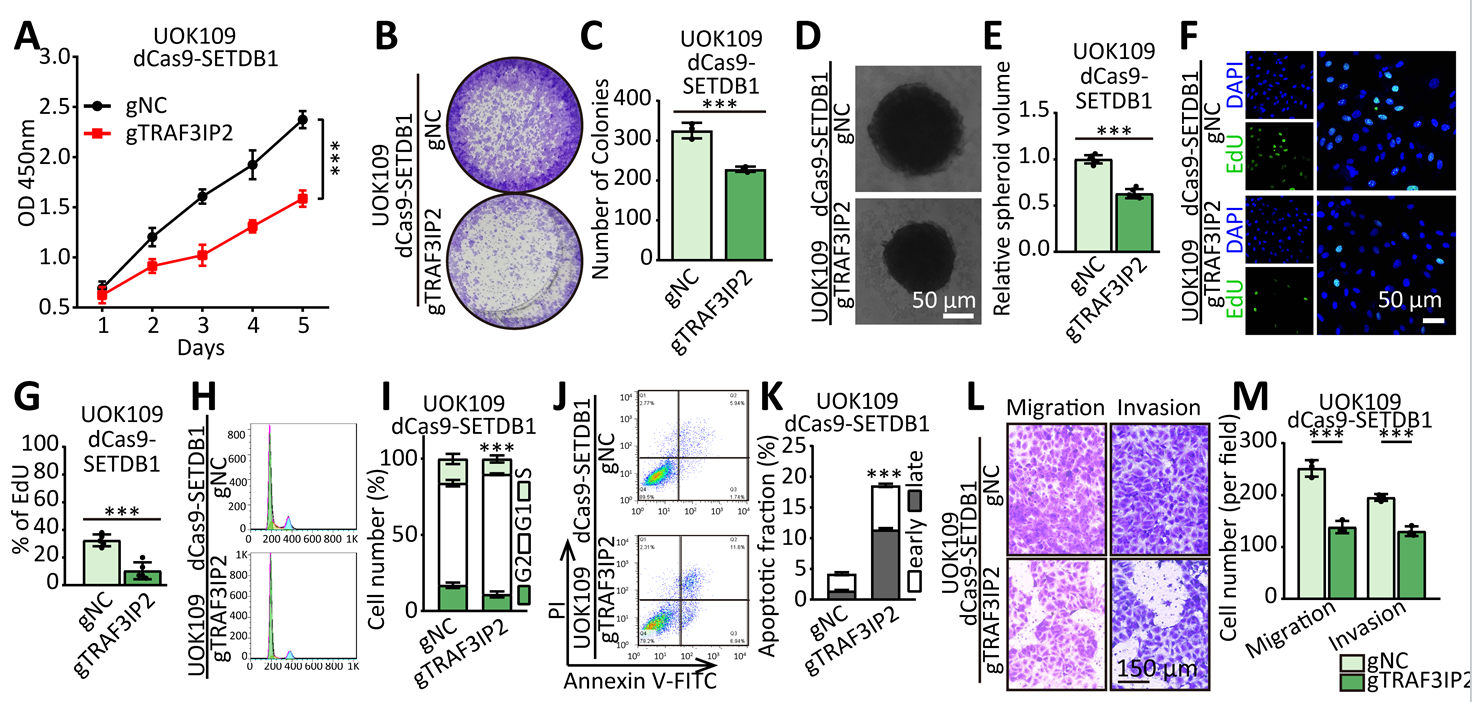


**Figure S7 SETDB1 inhibits development of *NONO-TFE3* tRCC by down-regulating the expression of TRAF3IP2. (A-E)** The effects of SETDB1 on the proliferation of UOK109 cells respectively were examined by CCK-8 assay **(A)**, colony formation assays **(B-C)** and tumor sphere formation **(D-E)**. **(F-G)** EdU assays were used to detect the proliferation rate of UOK109 cells after transfection for 48h. **(H-I)** Cell cycle was analyzed using flow cytometry after transfection for 48h. **(J-K)** Cell apoptosis was analyzed via flow cytometry using an Annexin V/PI kit after transfection for 48h. **(L-M)** Migration and invasion assays were performed with transfected cells using Transwell inserts. The data are presented as the mean ± SD, **P*< 0.05, ***P*< 0.01, ****P*< 0.001

**Supplementary Tables**

**Table S1.** Primers used for real-time PCR.

| Target  gene | Primer sequence (5’-3’) | | Size  (bp) |
| --- | --- | --- | --- |
|  | Forward | Reverse |  |
| TRAF3IP2-AS1 | TTTGGCGGCTATGCAGGATT | TGTCCATGTGGTATTGGGCA | 189 |
| TRAF3IP2 | TGGCACCCAACAGCTTGTC | GATACAGGCCGCTGGTGATTT | 106 |
| PSENEN | CTGGAGCGAGTGTCCAATGAG | GCGCCAGACATAGCCTTTGAT | 171 |
| VASN | TCTCACCTATCGCAACCTATCG | CAGACGGAGTAAGTGGCGTT | 117 |
| 18s rRNA | CAGCCACCCGAGATTGAGCA | TAGTAGCGACGGGCGGTGTG | 252 |
| HNRNPK | CAATGGTGAATTTGGTAAACGCC | GTAGTCTGTACGGAGAGCCTTA | 172 |
| DNMT1 | CCTAGCCCCAGGATTACAAGG | ACTCATCCGATTTGGCTCTTTC | 118 |
| SETDB1 | TAAGACTTGGCACAAAGGCAC | TCCCCGACAGTAGACTCTTTC | 104 |
| TFE3 | TGCCTGTGTCAGGGAATCTG | CGACGCTCAATTAGGTTGTGAT | 184 |
| IGF2BP2 | AGTGGAATTGCATGGGAAAATCA | CAACGGCGGTTTCTGTGTC | 185 |
| DGCR8 | AGCGTGAGCTTTACCGAGAG | CTACCCCGTCACCAACACTC | 157 |
| HNRNPC | CCCTTCTCCGTCCCCTCTAC | CCCGAGCAATAGGAGGAGGA | 122 |
| RBFOX2 | TTTAATGAACGTGGCTCTAAGGG | CGGACCATATACAGCTCCAAC | 216 |
| ELAVL1 | AACTACGTGACCGCGAAGG | CGCCCAAACCGAGAGAACA | 194 |
| FUS | ATGGCCTCAAACGATTATACCCA | GTAACTCTGCTGTCCGTAGGG | 114 |
| TARDBP | GGGAAATCTGGTGTATGTTGTCA | TTTTCTGGACTGCTCTTTTCACT | 104 |
| PRPF8 | TGACAAAAGGGTTTACTTGGGTG | CTCATTGACGAAGGAAATGGCT | 145 |
| MOV10 | GGGCCAGTGTTTCGAGAGTTT | TCTTGGTGACGTAGGCCAGA | 176 |
| UPF1 | ACCGACTTTACTCTTCCTAGCC | AGGTCCTTCGTGTAATAGGTGTC | 248 |
| SMNDC1 | AGCGGAGATTGAGGAGATAGATG | GGTTCAACAGTGGAGTCACTTC | 92 |
| U2AF2 | CGGCAGCTCAACGAGAATAAA | GGGAACGAATCAGTCCACCG | 214 |
| CSTF2T | TCACTGCGTTCCGTGTTCG | CCCTTGGGTTTTCCCGTCTC | 134 |
| SRSF1 | GGAAGACGCGGTGTATGGTC | CACCTGCTTCACGCATGTG | 236 |
| HNRNPA1 | TCAGAGTCTCCTAAAGAGCCC | ACCTTGTGTGGCCTTGCAT | 227 |
| GTF2F1 | CAACTTTGCTACGTGGAATCAGG | GAGGACGATGCCGTACTTCTT | 154 |
| TIA1 | CGAGATGCCCAAGACTCTATACG | CCTTACCCATTATCTTCCGTCCA | 215 |
| XRN2 | CCTTCGGCTTAATGTTCTTCGT | TGAAAACCCAGTCATCAATGCT | 104 |
| SRSF7 | CGGTACGGAGGAGAAACCAAG | AGCCACAAATCACCTTTCCATC | 208 |

**Table S2.** Primers used for ChIP/CHIRP/MS2-RIP assay.

| Target  promoter | Primer sequence (5’-3’) | | Size  (bp) |
| --- | --- | --- | --- |
|  | Forward | Reverse |  |
| PSENEN site1 | GCACCTTGACATAGACGTTGC | GATTCCACCCTGCCCGC | 250 |
| PSENEN site2 | GGTACAGGTTGAGCAGCACT | TCTTAGGGGCAAAGGATCGC | 167 |
| PSENEN site3 | AGAAGGGGAAGGGCCAGG | AAGGACAAGTGAGAACGGGC | 286 |
| VASN site1 | AGGCTGCACAGCACTGATTG | TGAAGGCACTGCTAACATTGAA | 242 |
| VASN site2 | TTATACTTAGGAGGCTCTTGAAAGG | GAATCCTAAGCGTCACAGCC | 296 |
| VASN site3 | GAAACCTGCCCAGTGTCATC | AGGCAGGTCGCGCCTTA | 216 |
| TRAF3IP2 primer1 | CAACGGCTGAGGAGTCACTT | CTTCTTAGCTGGGTGGCGAA | 110 |
| TRAF3IP2 primer2 | TCTACGGAGCCTAGGAAGCC | CACTGCGACTACACTGAGGT | 200 |
| HES1 | AAACCAAAGCCCAGAGGGAG | ATATCTGGGACTGCACGCGA | 240 |
| HEY1 | GCCTCGGTCCCAGAAATCAA | CCTAGCGCCACCGTAAATCT | 246 |
| MYC | ATAGCGATTGGTTGCTCCCC | CCCTCCACCACCTCCAAAAG | 230 |
| BCL2 | GGCGTCCCTAGACATTGCTT | CTGGACCCTTTCTAGCCGTG | 194 |
| CCND1 | ACTTACTGTTGGTGGGACGC | ATGAACAGGGGAGGGATGGA | 236 |

**Table S3.** Primary antibodies used in this study.

| Source | Primary antibodies | Catalog no. | Working dilution |
| --- | --- | --- | --- |
| Sigma-Aldrich | Anti-TFE3 antibody produced in rabbit | HPA023881 | WB: 1:2000 ChIP: 5μg |
| ABclonal | Anti-TRAF3IP2 antibody produced in rabbit | A6776 | WB: 1:1000 IHC: 1:200 |
| Santa Cruz Biotechnology | Anti-TRAF3IP2 antibody produced in mouse | sc-100647 | CoIP/ChIP: 5μg |
| ProteinTech | Anti-NOTCH1 antibody produced in rabbit | 10062-2-AP | WB: 1:1500 CoIP: 5μg |
| ProteinTech | Anti-Histone H3 antibody produced in rabbit | 17168-1-AP | WB: 1:1000 |
| Abcam | Anti-Histone H3 antibody produced in rabbit | ab1791 | ChIP: 5μg |
| Abcam | Anti-H3K9me3 antibody produced in rabbit | ab8898 | ChIP: 5μg |
| Abcam | Anti-5mC antibody produced in rabbit | ab214727 | MeDIP: 5μg |
| ProteinTech | Anti-HA antibody produced in rabbit | 51064-2-AP | WB: 1:2500 |
| ProteinTech | Anti-V5 antibody produced in rabbit | 14440-1-AP | WB: 1:2500 |
| ProteinTech | Anti-Flag antibody produced in rabbit | 80010-1-RR | WB: 1:2500 ChIP: 5μg |
| ProteinTech | Anti-GFP antibody produced in rabbit | 50430-2-AP | WB: 1:2500 RIP: 5μg |
| ProteinTech | Anti-KAT2B antibody produced in rabbit | 13983-1-AP | WB: 1:1000 |
| ProteinTech | Anti-MAML1 antibody produced in rabbit | 55493-1-AP | WB: 1:1000 |
| ABclonal | Anti-EP300 antibody produced in rabbit | A13016 | WB: 1:1000 |
| ProteinTech | Anti-RBPJ antibody produced in rabbit | 14613-1-AP | WB: 1:1000 CoIP: 5μg |
| Cell Signaling Technology | Anti-RBPJ antibody produced in rabbit | 5313 | ChIP: 5μg |
| ProteinTech | Anti-HEY1 antibody produced in rabbit | 19929-1-AP | WB: 1:1000 |
| ABclonal | Anti-HES1 antibody produced in rabbit | A11718 | WB: 1:1000 |
| ProteinTech | Anti-MYC antibody produced in rabbit | 10828-1-AP | WB: 1:1000 |
| ABclonal | Anti-PSENEN antibody produced in rabbit | A15172 | WB: 1:1000 |
| ABclonal | Anti-VASN antibody produced in rabbit | A16215 | WB: 1:1000 |
| ABclonal | Anti-NUMB antibody produced in rabbit | A9352 | WB: 1:1000 |
| ProteinTech | Anti-HNRNPK antibody produced in rabbit | 11426-1-AP | WB: 1:1000 RIP/CoIP/ChIP: 5μg |
| ProteinTech | Anti-DNMT1 antibody produced in rabbit | 24206-1-AP | WB: 1:1000 CoIP: 5μg |
| ProteinTech | Anti-SETDB1 antibody produced in rabbit | 11231-1-AP | WB: 1:1000 CoIP: 5μg |
| ABclonal | Anti-ACTB antibody produced in rabbit | AC026 | WB: 1:10000 |

**Table S4.** Primers used for MSP analysis.

| Target  transcript | Primer sequence (5’-3’) | | Size  (bp) |
| --- | --- | --- | --- |
|  | Forward | Reverse |  |
| TRAF3IP2 M 1 | TTTTGGTTAATAAGGTGAAATTTCG | GCCCAATCTAAAATACAATAACGAT | 173 |
| TRAF3IP2 U 1 | TTTGGTTAATAAGGTGAAATTTTGT | ACCCAATCTAAAATACAATAACAAT | 172 |
| TRAF3IP2 M 2 | TTTTGGTTAATAAGGTGAAATTTCG | GCCCAATCTAAAATACAATAACGAT | 173 |
| TRAF3IP2 U 2 | TTGGTTAATAAGGTGAAATTTTGT | ACCCAATCTAAAATACAATAACAAT | 171 |

**Table S5.** Small-molecule inhibitors used in this study.

| Source | Small-molecule inhibitors | Catalog no. | Working dilution |
| --- | --- | --- | --- |
| MedChemExpress | DAPT | HY-13027 | 20 µM |
| Selleck | 5-Azacytidine (5-Aza) | S1782 | 10 µM |
| Selleck | Bobcat339 | S6682 | 10 μM |
| Selleck | BRD4770 | S7591 | 2.5 μM |
| MedChemExpress | Toxoflavin | HY-100760 | 1 μM |

**Table S6.** SiRNA, shRNA and ASOs used for silencing target genes.

| Target  transcript | | Sequence (5’-3’) | |
| --- | --- | --- | --- |
|  |  | sense | antisense |
| TRAF3IP2-AS1 siRNA seq1 | | CAUUAGAAGUUAUAAUAAACA | UUUAUUAUAACUUCUAAUGUG |
| TRAF3IP2-AS1 siRNA seq2 | | GGAGUAUGUGCUUAAGGAAAU | UUCCUUAAGCACAUACUCCAA |
| TRAF3IP2-AS1 siRNA seq3 | | GAGCUGUGAUUCAAAUAUAAG | UAUAUUUGAAUCACAGCUCUG |
| TFE3 shRNA | CAGCTCCGAATTCAGGAACTA | | |
| TRAF3IP2 shRNA | CCGTGATGATAATCGTAGCAA | | |
| HNRNPK shRNA | TGATGTTTGATGACCGTCGCG | | |
| DNMT1 shRNA | GCCCAATGAGACTGACATCAA | | |
| SETDB1 shRNA | GCTCAGATGATAACTTCTGTA | | |
| IGF2BP2 shRNA | AGTGAAGCTGGAAGCGCATAT | | |
| DGCR8 shRNA | GCTCGATGAGTTAGAAGATTT | | |
| HNRNPC shRNA | GCCTTCGTTCAGTATGTTAAT | | |
| RBFOX2 shRNA | GTATATGGTCCGGAGTTATAT | | |
| ELAVL1 shRNA | GCAGCATTGGTGAAGTTGAAT | | |
| FUS shRNA | ATGAATGCAACCAGTGTAAGG | | |
| TARDBP shRNA | GCTCTAATTCTGGTGCAGCAA | | |
| PRPF8 shRNA | GCCCTGTATGTGTTACGTGAA | | |
| MOV10 shRNA | GCTGACCTTCAAGGTGAACTT | | |
| UPF1 shRNA | GCATCTTATTCTGGGTAATAA | | |
| SMNDC1 shRNA | CTGGTAAAGTTGGAGTAGGAA | | |
| U2AF2 shRNA | CGACGAGGAGTATGAGGAGAT | | |
| CSTF2T shRNA | GCGTCTGTTCACTTTAAGTTA | | |
| SRSF1 shRNA | GAAGCAGGTGATGTATGTTAT | | |
| HNRNPA1 shRNA | GACCAGGTGCCTACTTAACAG | | |
| GTF2F1 shRNA | GCTGTGACTTATCCACCACAT | | |
| TIA1 shRNA | GCCGTTGTTTACTTAAAGATT | | |
| XRN2 shRNA | CGTGAGTATTTGGAAAGAGAA | | |
| SRSF7 shRNA | GATCAAGATCCAGGTCTATTT | | |
| TRAF3IP2-AS1 ASO | G^M^G^M^A^M^G^M^T^D^A^D^T^D^G^D^T^D^G^D^C^D^T^D^T^D^A^D^A^D^G^D^G^M^A^M^A^M^A^M^ | | |

**Table S7.** Guide RNA used for CRISPR/dCas9 system.

| Target promoter | gRNA sequence (5’-3’) |
| --- | --- |
| TRAF3IP2-AS1 gRNA1 | AGGGGGAGAAAGCCCCTTCT |
| TRAF3IP2-AS1 gRNA2 | GGGATCGATGACTCGGCTGG |
| TRAF3IP2-AS1 gRNA3 | GGGGTGTGTGTGGCGAAGGG |
| TRAF3IP2 gRNA1 | CCGTTGATTCACTGCCCACA |
| TRAF3IP2 gRNA2 | TGGCCCTTTCTCATGAATGA |
| TRAF3IP2 gRNA3 | TCTGCTCAAAATCTCCCTGT |

**Table S8.** Primers used for MSP analysis.

| Target promoter | gRNA sequence (5’-3’) |
| --- | --- |
| SETDB1 Full-length F | gggagacccaagctggctagcGCCACCATGTCTTCCCTTCCTGGGTGC |
| SETDB1 Full-length R | tccttgtagtcacttaagctAAGAAGACGTCCTCTGCATTCAA |
| SETDB1 Δ1 F | gggagacccaagctggctagcGCCACCATGTCTTCCCTTCCTGGGTGC |
| SETDB1 Δ1 R | tccttgtagtcacttaagctACTGAGTGCAGGAGATGTAGGGG |
| SETDB1 Δ2 F | gggagacccaagctggctagcGCCACCATGTCTTCCCTTCCTGGGTGC |
| SETDB1 Δ2 R | tccttgtagtcacttaagctCTCATTGACACAGGATAGGGGAA |
| SETDB1 Δ3 F | gggagacccaagctggctagcGCCACCATGTCTTCCCTTCCTGGGTGC |
| SETDB1 Δ3 R | tccttgtagtcacttaagctTCCATGTTGCACCAACCGG |
| SETDB1 Δ4 F1 | gggagacccaagctggctagcATGTCTTCCCTTCCTGGGTGC |
| SETDB1 Δ4 R1 | agagtagctcTCCATGTTGCACCAACCGG |
| SETDB1 Δ4 F2 | gcaacatggaGAGCTACTCTGTTGCTGTGGGG |
| SETDB1 Δ4 R2 | ccggttggtgcaacatggaAAGAAGACGTCCTCTGCATTCA |
| HNRNPK Full-length F | gggagacccaagctggctagcGCCACCATGGAAACTGAACAGCCAGAAGA |
| HNRNPK Full-length R | tccttgtagtcacttaagctGAAAAACTTTCCAGAATACTGCTTCA |
| HNRNPK Δ1 F | gggagacccaagctggctagcGCCACCATGGAAACTGAACAGCCAGAAGA |
| HNRNPK Δ1 R | tccttgtagtcacttaagctAACCATGCCGTCGTAACGG |
| HNRNPK Δ2 F | gggagacccaagctggctagcGCCACCATGGAAACTGAACAGCCAGAAGA |
| HNRNPK Δ2 R | tccttgtagtcacttaagctGATGATCTTTATGCACTCTACAACCC |
| HNRNPK Δ3 F | gggagacccaagctggctagcGCCACCATGGAAACTGAACAGCCAGAAGA |
| HNRNPK Δ3 R | tccttgtagtcacttaagctGATTTTCTTCAGAATTTCTCCAATTG |
| HNRNPK Δ4 F | gggagacccaagctggctagcGCCACCATGATGTTTGATGACCGTCGC |
| HNRNPK Δ4 R | tccttgtagtcacttaagctAACCATGCCGTCGTAACGG |
| DNMT1 Full-length F | gggagacccaagctggctagcGCCACCATGCCGGCGCGTACCGCC |
| DNMT1 Full-length R | tccttgtagtcacttaagctGTCCTTAGCAGCTTCCTCCTCC |
| DNMT1 Δ1 F | gggagacccaagctggctagcGCCACCATGCCGGCGCGTACCGCC |
| DNMT1 Δ1 R | tccttgtagtcacttaagctATTTACTTTTTCAGGTTCTTTTTCTTCG |
| DNMT1 Δ2 F | gggagacccaagctggctagcGCCACCATGCCACAGATTTCTGATGAAAAAGACG |
| DNMT1 Δ2 R | tccttgtagtcacttaagctGAAGATCTGGTAGACCAGCTTGGT |
| DNMT1 Δ3 F | gggagacccaagctggctagcGCCACCATGGATACTTTCTTCGCAGAGCAAATTG |
| DNMT1 Δ3 R | tccttgtagtcacttaagctCCCTTTGTTTCCAGGGCTACG |
| DNMT1 Δ4 F | gggagacccaagctggctagcGCCACCATGCAAGCCTGTGAGCCGAGCG |
| DNMT1 Δ4 R | tccttgtagtcacttaagctGTCCTTAGCAGCTTCCTCCTCC |
| DNMT1 Δ5 F | gggagacccaagctggctagcGCCACCATGCCGGCGCGTACCGCC |
| DNMT1 Δ5 R | tccttgtagtcacttaagctCCCTTTGTTTCCAGGGCTACG |
| TRAF3IP2-AS1 NheI | tttaaatttgcgcatgctagcCGGAAGGGGCGGCGGAGC |
| TRAF3IP2-AS1 HindIII | acgacggccagtgccaagcttTTTGAGGTTGTTCTCTAAATGTTTATTATAA |
| TRAF3IP2-AS1 HindIII 500 | acgacggccagtgccaagcttAAATATGGAGAAAGCCGACAGAA |
| TRAF3IP2-AS1 HindIII 1000 | acgacggccagtgccaagcttTATCTCTGCAGGGGAAATTCTTAGA |
| TRAF3IP2-AS1 HindIII 1500 | acgacggccagtgccaagcttGCGGTGTCATGAGTTCCTCATT |
| TRAF3IP2-AS1 HindIII 2000 | acgacggccagtgccaagcttGAGGAGGCAAAAAGGAAGACAA |
| TRAF3IP2-AS1 HindIII 2500 | acgacggccagtgccaagcttGCCACTGTTCACTGTGGATGTG |
| TRAF3IP2-AS1 NheI 2500 | tttaaatttgcgcatgctagcTTCTGCCTTCCAGATGGGC |
| TRAF3IP2-AS1 NheI 3000 | tttaaatttgcgcatgctagcATCTCGTTTGGTCCTTAAAACAAGC |
| TRAF3IP2-AS1 NheI 3500 | tttaaatttgcgcatgctagcATGGTGACTACGTGGGAAATGC |
| TRAF3IP2-AS1 NheI 4000 | tttaaatttgcgcatgctagcTAAATTTTAATCATTAACTGAATTCAAAATAA |
| TRAF3IP2-AS1 NheI 4500 | tttaaatttgcgcatgctagcGGATTTAAAAAAAAATCTTTAAACTCATTAA |
| AS1 AS NheI | acgacggccagtgccaagcttCGGAAGGGGCGGCGGAGC |
| AS1 AS HindIII | tttaaatttgcgcatgctagcTTTGAGGTTGTTCTCTAAATGTTTATTATAA |
|  |  |
